# Decoding the Oxygen Reduction Reaction: Mechanistic Insights from Transition Metal Heterostructures

**DOI:** 10.1007/s40820-026-02197-6

**Published:** 2026-05-04

**Authors:** Mingyu Sun, Xiayan Zhang, Jia Wang, Jialu Liu, Jinhai He, Wanmiao Ge, Shengwei Kong, Guoqing Zhang, Mai Gao, Jingqiang Wang, Zixu Sun, Yaping Yan, Xinjian Shi, Yao Xiao

**Affiliations:** 1 National & Local Joint Engineering Research Center for Applied Technology of Hybrid Nanomaterials, School of Nanoscience and Materials Engineering, Henan University, Kaifeng, 475004 People’s Republic of China; 2 College of Chemistry and Chemical Engineering, Inner Mongolia University, Hohhot, 010021 Inner Mongolia People’s Republic of China; 3 Henan Engineering Technology Research Center for Fiber Preparation and Modification, Henan University of Engineering, Zhengzhou, 451191 People’s Republic of China; 4 College of Chemistry and Materials Engineering, Wenzhou University, Wenzhou, 325035 People’s Republic of China

**Keywords:** Oxygen reduction reaction, Transition metal catalysts, Heterogeneous electrocatalysis

## Abstract

The oxygen reduction reaction (ORR) plays a central role in determining the efficiency and durability of a broad range of electrochemical energy conversion technologies, while its intrinsically slow kinetics and multistep reaction nature remain key challenges. Transition metal-based heterogeneous electrocatalysts, particularly those incorporating Fe, Mn, Co, Ni, and Cu, have been extensively investigated as ORR active systems owing to their earth abundance, diverse coordination environments, and tunable electronic structures. Recent studies show that ORR on these catalysts can diverge into two-electron and four-electron pathways from a common O_2_ reactant, with pathway selectivity governed by atomic-scale coordination and electronic effects. This review summarizes current understanding of ORR mechanisms on transition metal-based catalysts, with a focus on how atomic-scale structural features are related to reaction activity, selectivity, and stability under different electrolyte conditions. Representative applications in metal–air batteries, fuel cells, and electrochemical hydrogen peroxide production are also discussed to illustrate how mechanistic insights are reflected in practical electrochemical systems. By distilling shared mechanistic features across different systems, this review provides a coherent framework for understanding and guiding ORR catalyst design.

## Highlights

- This review presents a unified mechanistic framework for oxygen reduction reaction catalysis across diverse transition metal systems (Fe, Mn, Co, Ni, Cu), linking electronic structure, coordination environment, and interfacial effects to pathway selectivity.
- In situ/operando techniques and interfacial engineering are emphasized as critical tools for atomic-level active site probing and performance optimization.
- A cross-scale design paradigm bridges molecular-level insights with practical applications in fuel cells, metal-air batteries, and H_2_O_2_ electrosynthesis.

## Graphical abstract

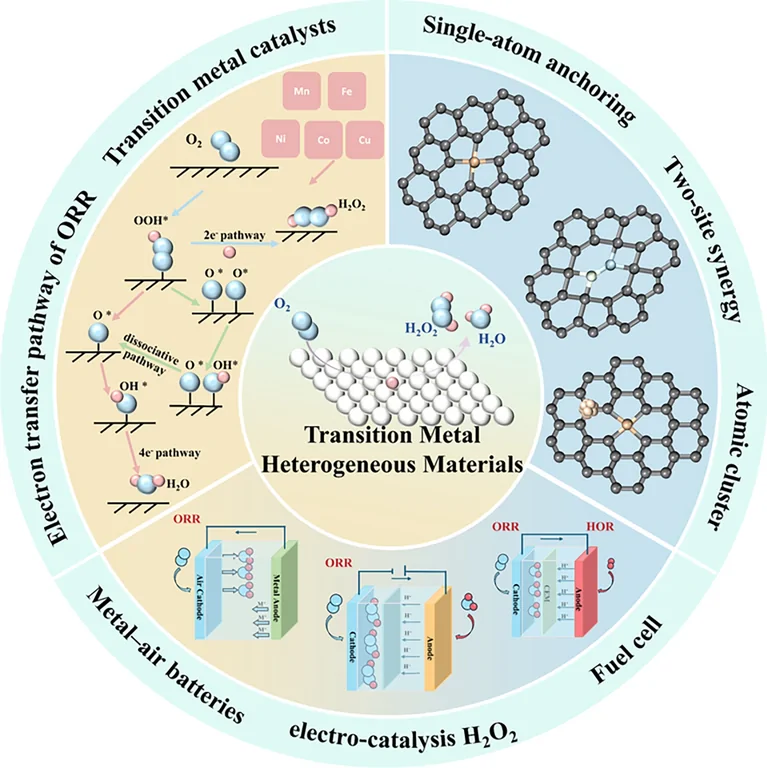

## Introduction

At a fundamental level, the oxygen reduction reaction (ORR) can be regarded as a highly coupled interfacial reaction network rather than a simple multielectron process proceeding along a single reaction coordinate. On electrode surfaces, the adsorption, activation, and reduction of molecular oxygen involve multiple near-degenerate intermediate states, including OOH*, O*, and OH*, whose formation, interconversion, and desorption compete with one another to determine the distribution of reaction flux among different pathways [1–3]. Because the free energy differences between these intermediates are often comparable in magnitude, the kinetic behavior of ORR exhibits pronounced sensitivity to local structural motifs, electrode potential, and interfacial environment, rendering the reaction system highly responsive to relatively small perturbations in both thermodynamic and kinetic terms [4–8]. As a prototypical proton-coupled electron transfer (PCET) reaction, the ORR reaction coordinate is not only governed by electronic structure but also modulated by interfacial electric fields, solvation structures, and proton donor configurations [9–12]. These factors can substantially alter charge distributions and reorganization energies at the transition states, thereby introducing strong potential dependence and environmental sensitivity. Consequently, the kinetic bottleneck of ORR is not confined to a single elementary step but can migrate among multiple steps under different operating conditions, manifesting as pathway competition, rearrangement of rate-determining steps, and environment-dependent shifts in reaction selectivity [13–15]. This intrinsic coupling of multiple pathways and length scales renders ORR difficult to describe adequately using a single energetic descriptor or static reaction model [16, 17].

Transition metal-based catalytic systems display distinct and complex behaviors in ORR studies, arising from the highly tunable coupling between the d electron structure of metal centers and their coordination environments. Metal–ligand interactions, mediated through crystal field splitting, orbital hybridization, and covalency modulation, reshape the bonding characteristics and charge transfer properties between metal centers and oxygen-containing intermediates, thereby influencing O–O bond activation, OOH* stability, and the preference of subsequent reaction pathways [18–20]. In contrast to the relatively rigid structures of noble metal surfaces, transition metal active sites are commonly embedded in heterogeneous coordination environments, where coordination number, ligand identity, and second coordination shell effects can modify the local electronic density of states at the atomic scale [21–24]. Under electrochemical operating conditions, applied potential, adsorbate coverage, and solvation collectively determine the effective oxidation state of the metal center, rendering both valence state and active site identity condition-dependent rather than static structural parameters [25–28]. In this context, ORR catalytic behavior no longer follows a single electronic structure descriptor but instead reflects the projection of multiple degrees of freedom onto the reaction coordinate. Additional complexity arises from the dynamic evolution of transition metal sites during operation, including coordination restructuring, ligand exchange, and localized hydration or redox processes, which cause the real reaction interface to adopt time- and potential-dependent state distributions. These dynamic characteristics imply that structure–activity relationships must be understood within the complete cycle of site formation, site stabilization, and site participation in catalysis, rather than inferred solely from idealized initial structures [29, 30].

In ORR catalysis, the concept of heterostructure extends beyond conventional bulk phase interfaces. At the atomic scale, heterogeneity in metal identity, coordination environment, and metal–support interactions can create localized junction-like regions with distinct electronic structures and adsorption behaviors [31–33]. From this perspective, single-atom and dual-atom catalysts may be regarded as atomic-scale heterostructures, in which asymmetric coordination or dissimilar neighboring atoms generate intrinsic electronic and chemical discontinuities analogous to classical interfacial effects. Such atomic-level heterogeneous motifs provide an effective platform for modulating intermediate stability, charge distribution, and reaction pathway branching in ORR [34–36].

**Fig. 1:**
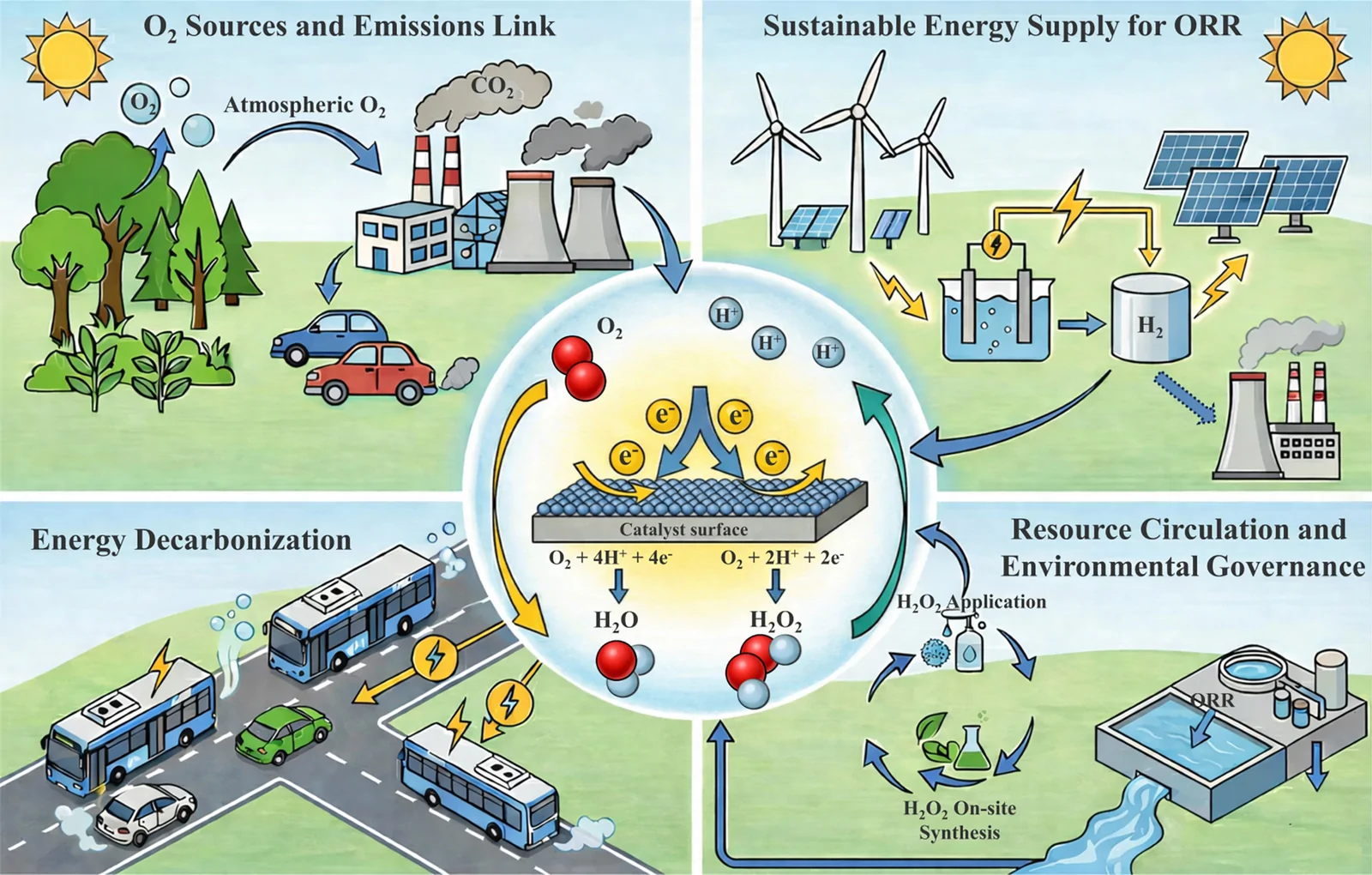
Schematic illustration of the sustainable energy-driven ORR

With continued advances in in situ and quasi-in situ characterization techniques, first-principles calculations, and microkinetic modeling, experimental and theoretical information concerning local structural evolution, electronic state rearrangement, and intermediate behavior during ORR has accumulated rapidly, enabling mechanistic discussions to progress from empirical correlations toward more structured analyses [37–39]. Nevertheless, these advances remain largely fragmented at the field level: Most studies are conducted within specific material systems, narrowly defined electrolyte compositions [40–42], or limited potential windows, and their mechanistic interpretations are often embedded within particular model assumptions, making it difficult to maintain consistent reference standards across different systems or operating conditions [43–46]. As a result, descriptions of rate-determining steps, key intermediate stabilities, or electronic structure regulation frequently differ across studies and may even appear superficially inconsistent. Such discrepancies do not necessarily reflect contradictions in the conclusions themselves but rather underscore the high sensitivity of ORR, as a multipathway and strongly interfacial-coupled reaction network, to experimental and environmental parameters [47–49]. In the absence of a unified conceptual coordinate system, single-point or system-specific mechanistic interpretations provide limited support for broader extrapolation and constrain the extraction of transferable insights from existing results [50–52]. This limitation is particularly pronounced in transition metal-based catalytic systems, where strong coupling among coordination structure, electronic state distribution, interfacial environment, and reaction pathway branching is intrinsic. Many studies address only one of these dimensions, thereby obscuring the collective effects that shape the overall reaction network [53, 54]. Under these circumstances, a mechanism-oriented integrative analysis of transition metal-based ORR catalysis becomes essential. This review aims to study the results scattered under different material systems and experimental conditions from a multidimensional framework including coordinated structure, electronic modulation, interface effects and reaction networks, in order to identify common control factors, describe the key uncertainties in the current understanding of mechanisms, clarify the boundaries of descriptor applicability, and construct an extensible conceptual structure. To support reasonable model development, experimental design and strategy formulation in complex catalytic systems (Fig. 1).

## Structure-Dependent Mechanisms of the ORR

Different transition metal-based ORR catalysts regulate reaction selectivity primarily by steering the divergence between the 2e^−^ and 4e^−^ pathways through distinct active site structures, oxygen adsorption configurations, intermediate evolution modes, and electron transfer characteristics [55–58]. Across diverse catalyst systems, the formation and subsequent fate of the OOH* intermediate constitute a universal mechanistic branching point that determines whether molecular oxygen undergoes partial reduction to H_2_O_2_ or complete reduction to H_2_O. The relative preference for the 2e^−^ or 4e^−^ pathway is therefore governed not by a single descriptor, but by the coupled regulation of O_2_ adsorption geometry, OOH* binding strength, O–O bond activation, and charge transfer capability at the active site [59–62].

**Fig. 2:**
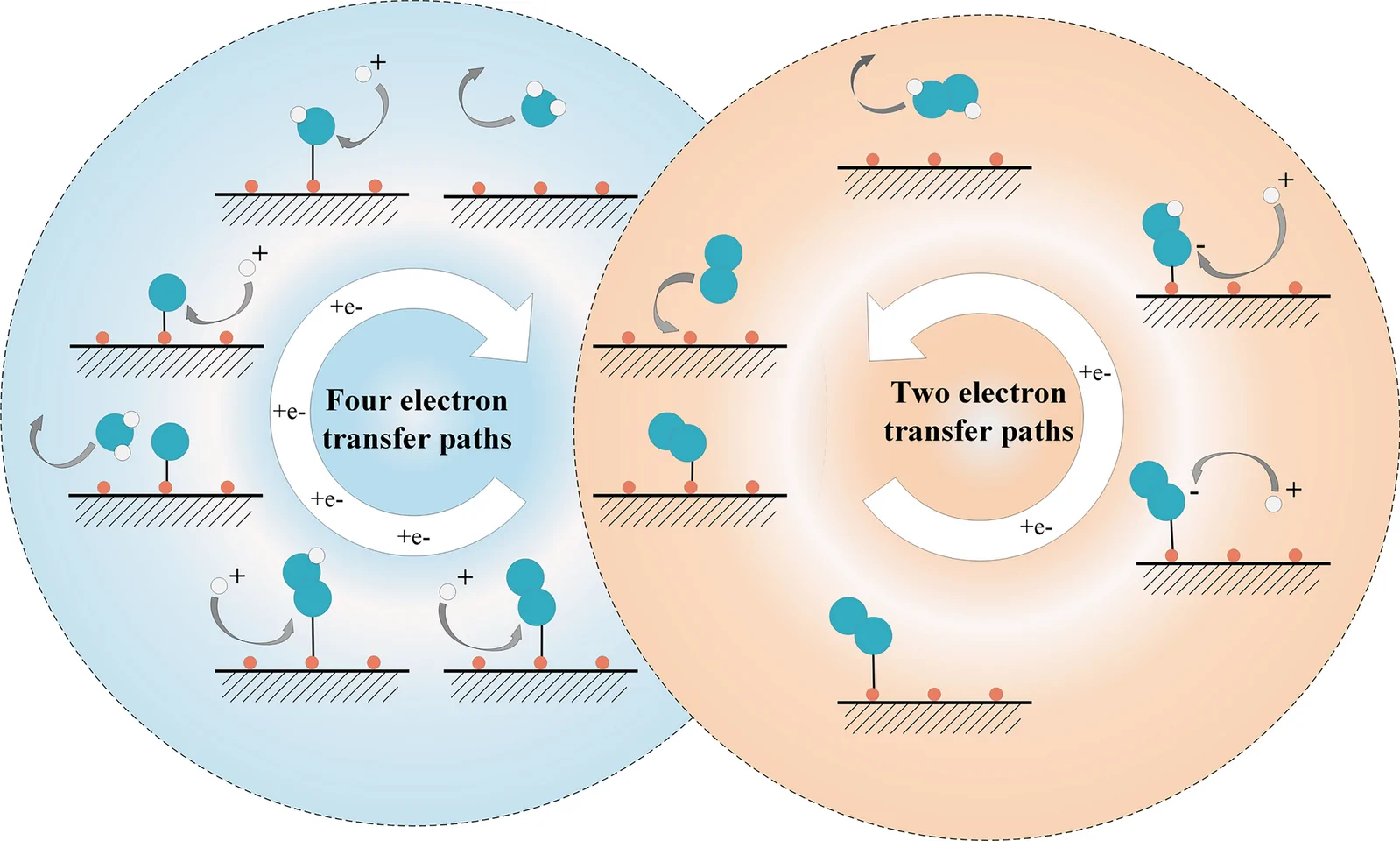
ORR mechanism diagram (orange represents the active sites of the catalyst, blue represents oxygen atoms, and white represents hydrogen atoms)

In single-atom catalysts (SACs), isolated metal centers—typically coordinated by nitrogen or oxygen atoms—promote end-on O_2_ adsorption and sequential proton–electron transfer at a single site [63–66]. In the absence of adjacent metal atoms, O–O bond cleavage depends entirely on the intrinsic ability of the metal center to stabilize and activate OOH*: weak OOH* binding favors direct desorption as H_2_O_2_ via the 2e^−^ pathway [67–69], whereas stronger OOH* adsorption enables O–O bond dissociation into O* and OH*, allowing the reaction to proceed through the 4e^−^ pathway toward H_2_O formation. In contrast, metal nanoparticle catalysts provide contiguous multiatom active sites that enable side-on or bridging O_2_ adsorption [70–72], significantly lowering the O–O bond dissociation barrier and favoring sustained multielectron transfer through the 4e^−^ pathway. For metal oxide catalysts, ORR proceeds via surface metal cations and lattice oxygen coordination, where limited electrical conductivity and metal–oxygen interaction strength critically influence pathway selection: Oxides with strong O_2_ activation capability (e.g., cobalt-based spinels) can approach the 4e^−^ pathway in alkaline media [73–76], whereas oxides with weaker O_2_ binding, such as TiO_2_, preferentially follow the 2e^−^ pathway to H_2_O_2_ formation (Fig. 2).

Across transition metal-based ORR catalysts, the intrinsic electronic structure of the metal center plays a decisive role in governing the divergence between the two-electron and four-electron pathways. Fe-based active sites generally exhibit strong interactions with oxygenated intermediates, particularly OOH*, which facilitate O–O bond activation and stabilize dissociated O* and OH* species, thereby lowering the energetic barrier for O–O bond cleavage and favoring the 4e^−^ reduction pathway to H_2_O [77–80]. Co-based catalysts often display intermediate and highly tunable behavior: their partially filled *d* orbitals enable effective O_2_ activation and OOH* stabilization, while the extent of O–O bond cleavage is strongly dependent on coordination environment and oxidation state, allowing Co sites to access either 4e^−^ or mixed pathways under different structural regulations. In contrast, Ni-based active sites typically bind OOH* more moderately and lack sufficient driving force for O–O bond dissociation, favoring OOH* desorption prior to bond cleavage and thus promoting selective 2e^−^ reduction to H_2_O_2_ [81–83]. Cu-based centers, characterized by more filled *d* orbitals and weaker interactions with oxygenated species, further suppress O–O bond activation and are intrinsically inclined toward end-on O_2_ adsorption and OOH* release, reinforcing their preference for the 2e^−^ pathway [84–86]. Mn-based catalysts, on the other hand, exhibit pronounced sensitivity to spin configuration and spin-dependent electron transfer, as the multivalent and spin-flexible nature of Mn centers strongly couples oxygen activation with spin state transitions, leading to ORR activity and selectivity that are highly dependent on coordination symmetry, ligand field strength, and operating conditions.

Beyond individual elements, the distinct behaviors of Fe-, Co-, Ni-, Cu-, and Mn-based catalysts reflect a set of universally applicable mechanistic principles that extend across diverse ORR systems. At a fundamental level, ORR pathway selection is governed by the balance between OOH stabilization and O–O bond activation, which can be continuously tuned through electronic structure modulation, multicenter cooperation, and interfacial charge redistribution [87–90]. Catalyst architectures that enable cooperative adsorption—such as adjacent metal sites, metal–nonmetal bonded ensembles, or metal–oxide interfaces—effectively lower O–O bond dissociation barriers and favor sustained multielectron transfer, exhibiting Fe or Co-like characteristics associated with the 4e^−^ pathway [91–94]. In contrast, isolated or weakly interacting sites that stabilize OOH* without promoting bond cleavage emulate Ni or Cu-like behavior and preferentially yield H_2_O_2_ via the 2e^−^ pathway. Spin-sensitive centers, exemplified by Mn-based systems, further highlight the role of electronic degeneracy and dynamic state evolution in shaping ORR kinetics. Collectively, these insights demonstrate that ORR selectivity across different materials is dictated not by catalyst class alone, but by shared and transferable mechanistic control factors, providing a unified framework for rational ORR catalyst design.

## ORR Mechanism over Transition Metal Heterogeneous Catalysts

**Fig. 3:**
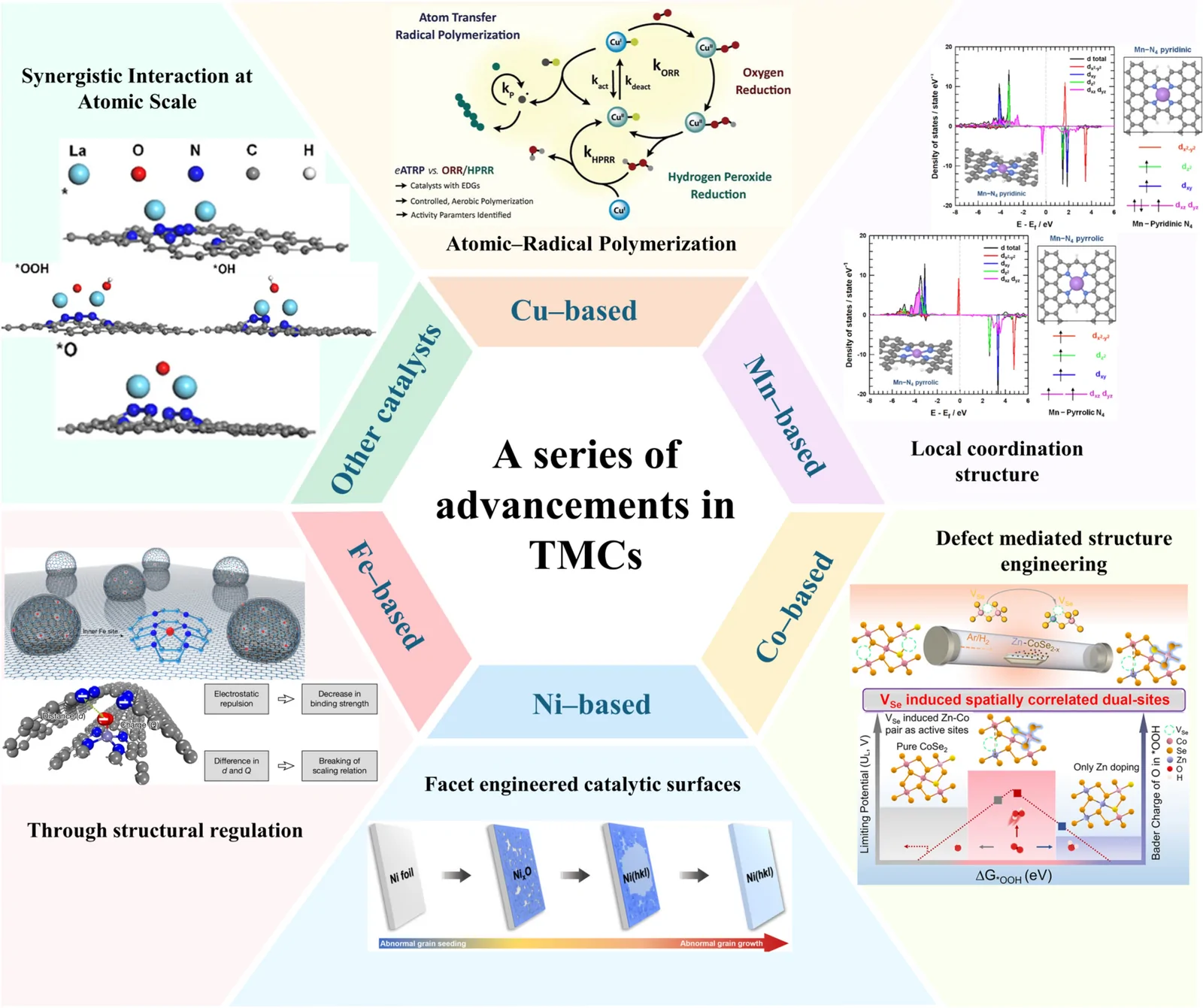
Representative advances in TMCs enabled by atomic-scale regulation and structural engineering [100–104, 125]. Copyright 2025, Wiley–VCH; Copyright 2025, Elsevier; Copyright 2025, Springer Nature; Copyright 2025 American Chemical Society

In addressing the catalytic requirements of the ORR, a diverse array of catalyst systems has been developed, encompassing precious metal-based, carbon-based, and transition metal-based catalysts (TMCs) [95–97]. Among these, heterogeneous electrocatalysis systems incorporating earth-abundant elements such as Fe, Mn, Co, Ni, and Cu have gained prominence as a primary research focus, owing to their compelling economic feasibility, environmental sustainability, and structural versatility [98, 99]. These TMCs have demonstrated notable advantages in electrocatalytic performance, positioning them as increasingly viable alternatives to precious metal-based benchmarks. To further optimize their performance, it is essential to develop a thorough comprehension of the structure–activity relationship, which not only elucidates the fundamental mechanisms of the ORR but also provides a critical theoretical and practical foundation for the rational design of novel, high-efficiency electrocatalysts (Fig. 3).

### Fe-Based Catalysts

Fe-based catalysts are widely regarded as promising alternatives to precious metal catalysts due to their high O_2_ activation capability and tunable electronic structure. Nevertheless, such systems face several challenges in the ORR [105–108]. Conventional Fe-based catalysts often exhibit limited activity, largely attributable to their constrained electronic configuration, which complicates the optimization of intermediate adsorption without compromising stability. Under electrochemical conditions [109, 110], the active iron sites are susceptible to structural reconstruction or metal leaching, leading to gradual degradation of catalytic durability. Additionally, the poisoning of iron centers in certain reaction media remains a non-negligible issue. Furthermore, the structural simplicity of these catalytic sites restricts the regulation of electron transfer pathways, making precise control over ORR kinetics difficult to achieve [111–113].

**Fig. 4:**
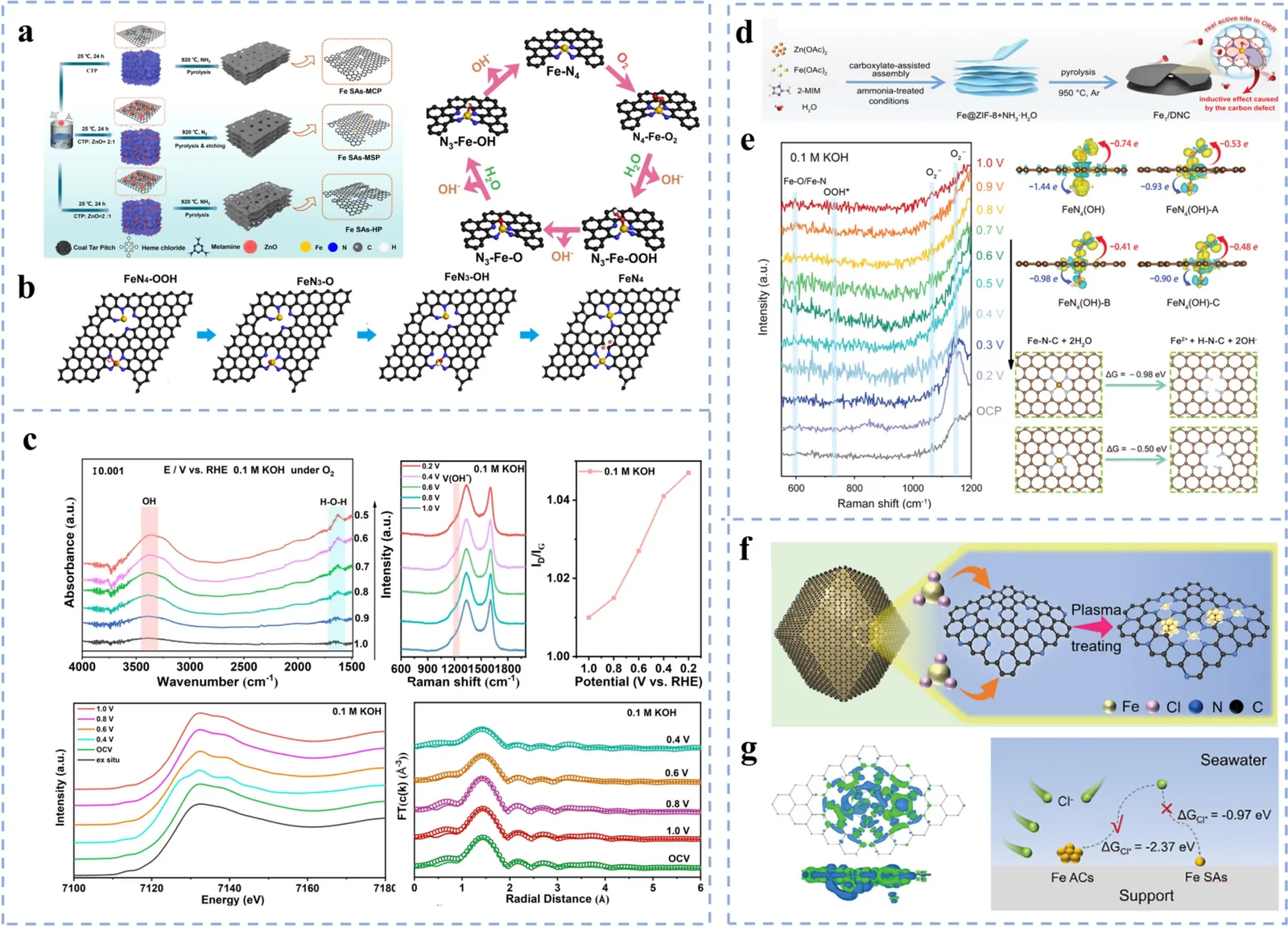
**a** Preparation process of Fe SAs–HP and its dynamic evolution mechanism under alkaline conditions. **b** Stepwise reduction process of Fe–N_4_ active center under alkaline conditions. **c** Fe SAs–HP was characterized in 0.1 M KOH using in situ ATR–SEIRAS, Raman spectroscopy, XANES, and FT–EXAFS [114]. Copyright 2024, Springer Nature. **d** Schematic diagram of the synthesis of Fe_1_/DNC [115]. Copyright 2024, Wiley–VCH. **e** In situ Raman spectroscopy, charge distribution and Fe site stability analysis in O_2_-saturated 0.1 M KOH. **f** Schematic diagram of the construction of Fe–N–C active sites by plasma treatment. **g** Differential charge density distribution between Fe_6_ clusters and single-atom Fe and the comparative mechanism of their resistance to Cl^−^ in seawater [116]. Copyright 2024, Wiley–VCH

Fe-based catalysts are a promising type of non-precious metal electrocatalysts for the ORR and show high activity in both acidic and alkaline environments because of high atomic utilization, clear Fe–N_x_ coordination structures, and adjustable electronic properties. Zhang et al. [114] developed a single-atom iron catalyst with a hierarchically porous structure using an embedding–pyrolysis–evaporation method (Fig. 4a). The porous and heterogeneous structure improved electronic regulation and oxygen adsorption at Fe active sites and also increased catalyst stability. In situ studies showed that Fe–N_4_ sites change their structure under alkaline conditions and form Fe–N_4_–OOH intermediates (Fig. 4b). This structural change improves the adsorption of oxygen species and supports O–O bond cleavage, which favors the 4e^−^ ORR pathway. Attenuated total reflection surface-enhanced infrared absorption spectroscopy (ATR–SEIRAS) and Raman spectroscopy (Fig. 4c) showed gradual accumulation of OH* species at Fe sites with little desorption. This result indicates strong intermediate binding and local structural distortion at Fe centers. X-ray absorption near edge structure (XANES) and X-ray absorption fine structure (EXAFS) results showed a decrease in Fe coordination number and oxidation state and support a transition from Fe–N_4_ to Fe–N_3_. This dynamic coordination change helps maintain active Fe sites during operation and explains the stable high ORR activity. Overall, these results show that Fe-based single-atom catalysts favor the 4e^−^ ORR pathway when strong OOH* binding is combined with flexible and stable Fe–N_x_ coordination structures.

In a complementary study, Ji et al. [115] constructed a single-atom Fe catalyst on a defect-rich N-doped carbon support using microenvironment engineering (Fig. 4d). The defect-induced Fe–N_4_(OH)–A structure improves electron transfer and strengthens the interaction between Fe active sites and reaction intermediates, while in situ Raman spectroscopy confirms stable adsorption and efficient conversion of key species such as OOH* and O_2_* at these sites. Free energy calculations show that the defect structure improves site anchoring and structural stability (Fig. 4e). These results indicate that defect engineering creates a more stable local environment for Fe single-atom sites and maintains strong intermediate binding during the ORR, which supports O–O bond cleavage and favors the 4e^−^ ORR pathway. Engineering the local microenvironment and defect structure provides a powerful means to concurrently boost the activity and durability of Fe-based single-atom ORR catalysts, resulting in ORR performance superior to commercial Pt/C in acidic, alkaline, and neutral media and revealing substantial potential for practical applications.

**Fig. 5:**
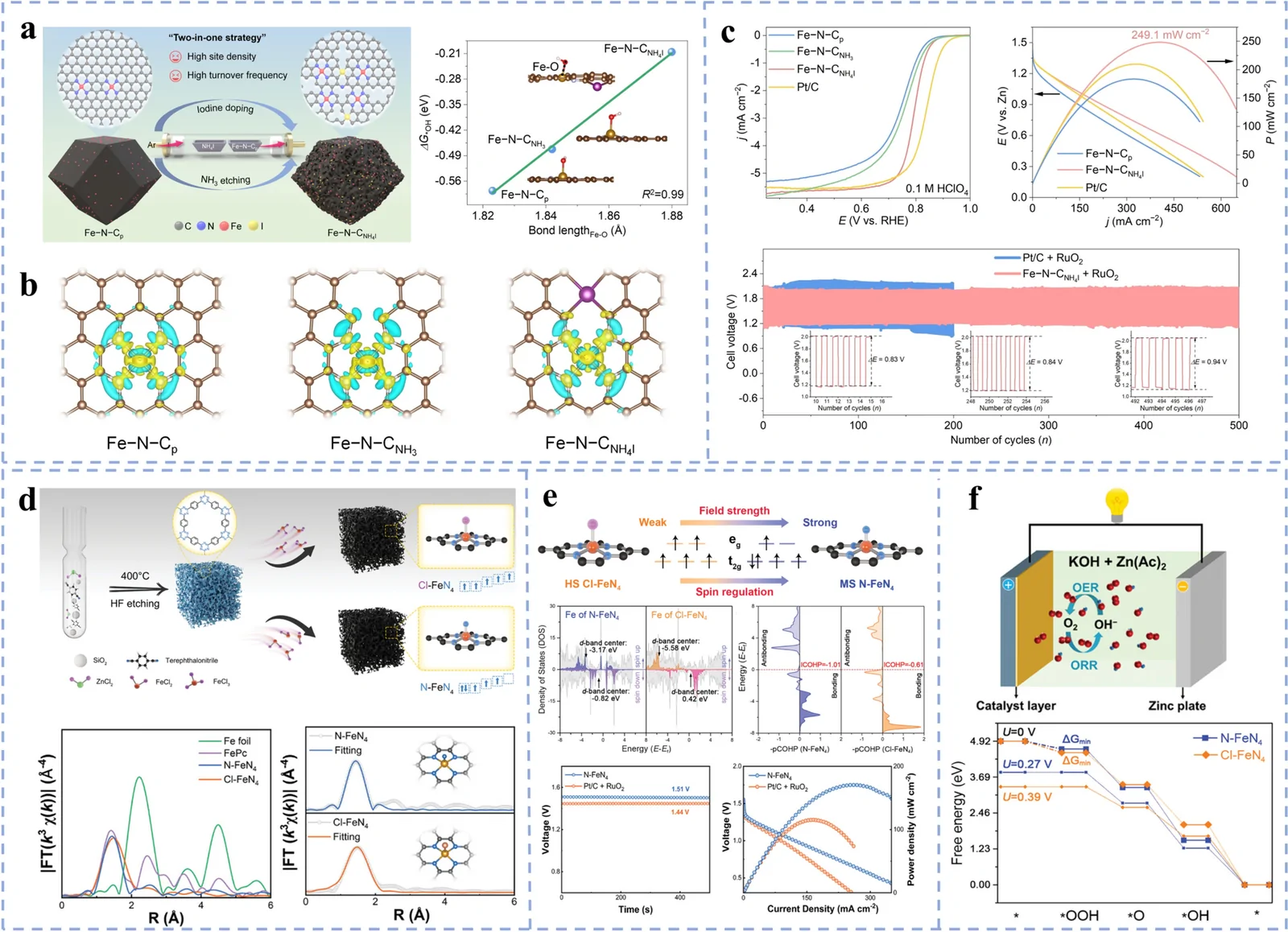
**a** Construction strategy of Fe–N–C_NH4I_ catalyst and its correlation with Fe–O bond length and ΔG_OH*_. **b** Charge density difference distribution under different coordination environments. **c** Comparison of ORR performance of each catalyst in acidic medium [117]. Copyright 2025, Wiley–VCH. **d** Synthesis schematic of N–FeN_4_ and Cl–FeN_4_ and comparison of their EXAFS characterization. **e** Differences between the two in atomic structure, electronic state density, Fe–OH bond strength and ORR reaction path. **f** Schematic diagram of liquid zinc–air battery [118]. Copyright 2024, Wiley–VCH

To enhance the practical performance of Fe single-atom catalysts under complex electrolyte conditions, recent studies have shifted the focus from isolated active sites to multisite synergy and interfacial electronic regulation. Rao et al. [116] reported a Fe single-atom catalyst modified with Fe clusters by plasma treatment (Fig. 4f), forming a cooperative structure between atomic sites and metallic clusters. The catalyst showed high ORR activity and stable operation in alkaline seawater electrolyte. Fe clusters donate electrons to neighboring Fe–N_4_ sites **(**Fig. 4g**)**, adjusting local charge distribution and suppressing active site poisoning through preferential Cl^−^ adsorption, which improves durability in chlorine-rich environments. To increase the accessibility of Fe active centers and tune their coordination geometry, Liu et al. [117] used NH_4_I-induced etching combined with iodine doping (Fig. 5a) to prepare Fe–N–C catalysts. The material showed elongated Fe–O bonds and a reduced ΔG_OH*_, which facilitates intermediate desorption and accelerates ORR kinetics. In zinc–air batteries, the catalyst delivered a peak power density of 249.1 mW cm^−2^ and long-term cycling stability (Fig. 5b, c), confirming its practical relevance. Qiao et al. [118] designed axial ligand-modified single-atom catalysts, Cl–FeN_4_ and N–FeN_4_, using chloride and pyridine nitrogen ligands (Fig. 5d). EXAFS analysis verified Fe–N_4_Cl and Fe–N_5_ coordination environments. Stronger ligand fields drive the Fe center from a high-spin to a medium-spin state (Fig. 5e). The shifted d band center in N–FeN_4_ strengthens OH* adsorption and lowers the ORR energy barrier. The catalysts were applied in rechargeable zinc–air batteries (Fig. 5f), demonstrating the effectiveness of axial ligand-controlled spin regulation.

**Fig. 6:**
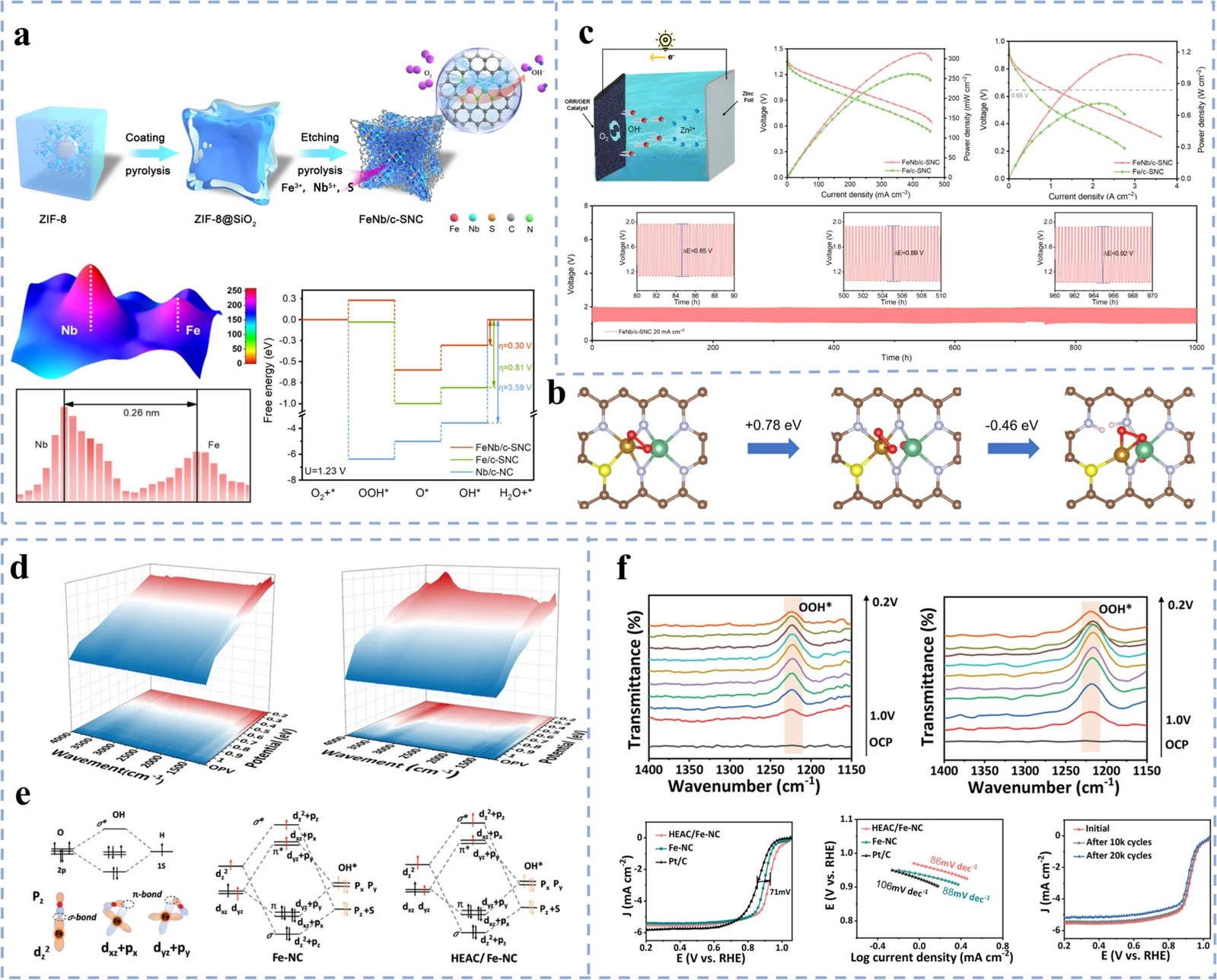
**a** Synthesis process, atomic distribution and free energy diagram of FeNb/c–SNC. **b** Structural evolution during demetallization. **c** Zn–air battery and its polarization curve, power density and long cycle performance [119]. Copyright 2024, American Chemical Society. **d** 3D in situ ATR–SEIRAS spectra of HEAC/Fe–NC and Fe–NC. **e** Possible orbital interactions between Fe centers and OH intermediates and their regulation on adsorption behavior. **f** Comparison of OH and OOH* band intensities of HEAC/Fe–NC and Fe–NC, as well as electrochemical tests of different samples [120]. Copyright 2024, Wiley–VCH

Sui’s team reported a FeNb/C–SNC catalyst with asymmetric diatomic sites prepared by template-assisted pyrolysis [119]. Fe and Nb atoms are co-embedded in a porous carbon matrix, forming Fe–S_1_N_3_ and Nb–N_4_ coordination structures at adjacent sites (Fig. 6a). This asymmetric diatomic configuration enables direct electronic interaction between Fe and Nb centers. Nb incorporation lowers the OH* adsorption energy on Fe sites and tunes the ORR pathway. Compared with single-metal catalysts, the bimetallic system shows improved intermediate stabilization and electron transfer (Fig. 6b). The free energy of OH* desorption decreases from + 0.78 to + 0.46 eV, reducing the reaction barrier. Zinc–air batteries assembled with this catalyst exhibit higher open-circuit voltage and power density (Fig. 6c) and stable operation for over 1000 h at 20 mA cm^−2^, indicating good structural durability. Yang et al. [120] combined high-entropy alloy catalysts (HEACs) with Fe–N_4_ sites to construct ORR catalysts with high activity and stability. In situ ATR–SEIRAS measurements show stronger OH* and OOH* signals during potential scanning (Fig. 6d), suggesting promoted formation and conversion of oxygen intermediates. Electronic structure analysis based on crystal field and molecular orbital models indicates that HEAC incorporation reorganizes the Fe *d* orbitals and weakens Fe–OH* bonding (Fig. 6e). Infrared and electrochemical results further confirm enhanced intermediate desorption, structural stability, and reaction kinetics in the HEAC/Fe–NC system (Fig. 6f), highlighting the role of high-entropy components in electronic modulation and ORR performance improvement.

With the advancement of research, the conventional Fe–N_4_ configuration has revealed several critical limitations. Under complex practical conditions—such as chloride-rich environments, elevated temperatures, or prolonged operation—the active sites are prone to migration, structural reconstruction, and potential deactivation, which compromises their stability [121]. Additionally, challenges remain in achieving high Fe atom loading density, structural uniformity across sites, and controllable synthesis. To address these issues, current research prioritizes the dual objectives of “high activity” and “enhanced stability,” and has developed multidimensional and complementary structural regulation strategies. At the synthesis level, in situ analysis of the pyrolysis process, the introduction of vapor deposition techniques, and the characterization of multiscale dynamic evolution mechanisms have collectively elucidated the intrinsic “synthesis–structure–performance” relationship within Fe/N/C systems [122]. From a holistic catalytic performance perspective, the “two-in-one” strategy—integrating high site density with high turnover frequency—is emerging as a key pathway to improve mass activity and the practical output performance of devices. Fe-based catalysts are currently at a critical transition stage, moving from “single-active-site construction” toward “collaborative interface design and controllable modulation.” Continuous performance enhancement relies not only on structural optimization but also on a comprehensive understanding of reaction mechanisms and site dynamic behavior. Future research should focus on strengthening in situ and quasi-in situ characterization methods, developing platforms that integrate multiscale simulation with experimental validation, and advancing the verification of long-term stable catalyst operation in real energy conversion devices.

### Mn-Based Catalysts

The application of Mn-based catalysts in the ORR shows considerable potential, particularly in alkaline environments. These catalysts demonstrate high stability, environmental compatibility, excellent corrosion resistance, and low oxygen evolution tendency, rendering them suitable for electrochemical systems requiring long-term operational reliability [101]. However, the 3*d*^5^ high-spin electronic configuration of manganese leads to weak adsorption capacity for O_2_ and key intermediates, often favoring the less efficient 2e^−^ pathway. This electronic structure presents challenges for achieving a highly selective 4e^−^ reduction process [123]. Furthermore, the formation of inert Mn–O bonds on the carbon support surface can impede the construction and maintenance of efficient active sites, thereby limiting intrinsic activity and overall catalytic performance. Current research primarily focuses on enhancing O_2_ adsorption and activation capabilities through electronic structure modulation and local coordination environment engineering. Key strategies include spin state regulation, construction of low-coordination structures, and introduction of heterogeneous interface synergy effects. These approaches aim to improve both the activity and stability of manganese-based ORR catalysts, advancing their development as high-performance non-precious metal electrocatalysts [124].

**Fig. 7:**
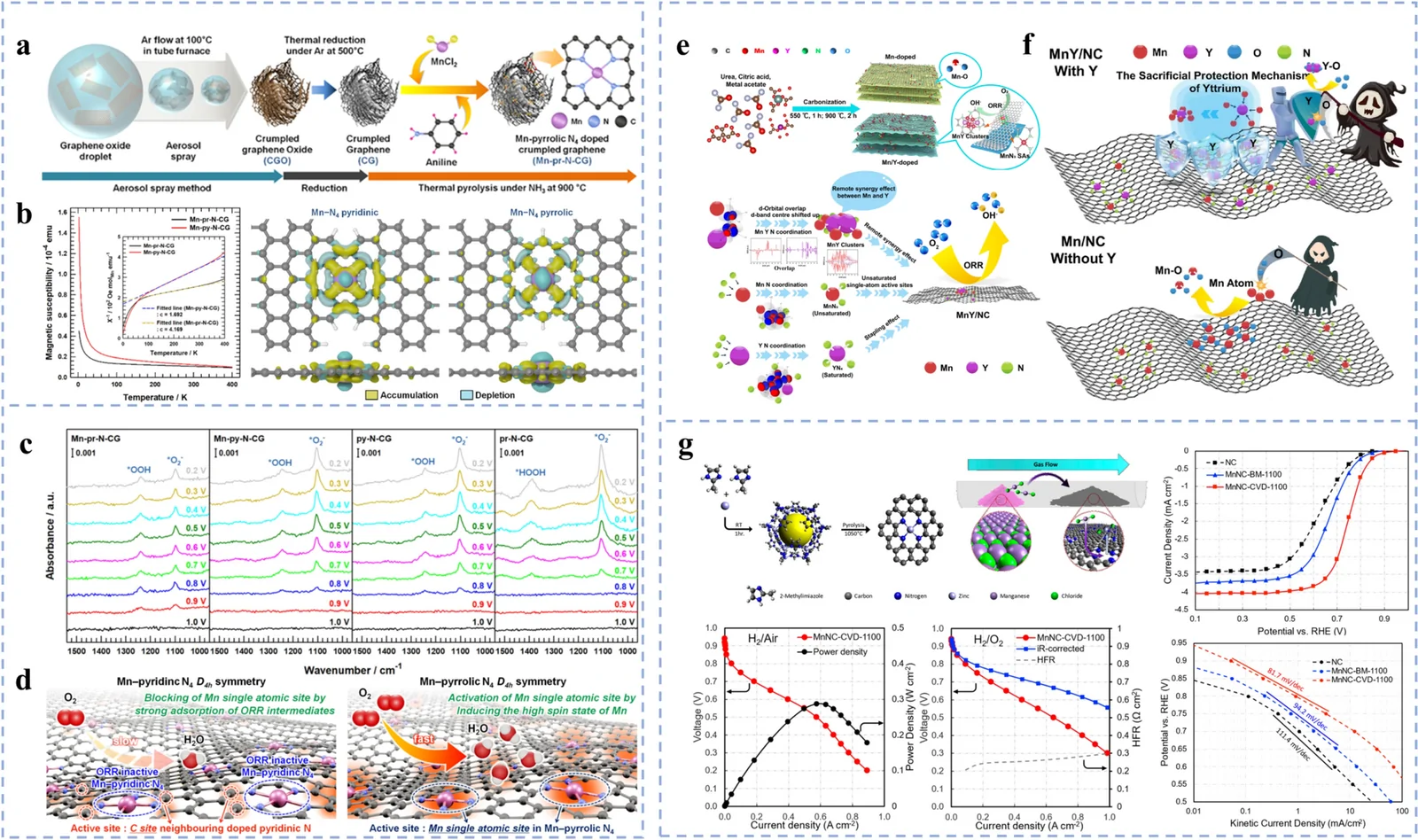
**a** Synthesis route of Mn–pr–N–CG. **b** Magnetization behavior and charge density difference of Mn–N_4_ (pyridine-type and pyrrole-type) coordination environment. **c** ATR–SEIRAS spectrum. **d** Different mechanism of activity source between Mn–py–N–CG and Mn–pr–N–CG in ORR [125]. Copyright 2024, American Chemical Society. **e** Schematic diagram of the synthesis of MnY/NC and the long-range synergistic effect mechanism of the catalyst. **f** Sacrificial protection mechanism of Y [126]. Copyright 2025, Elsevier. **g** Schematic diagram of the synthesis of MnNC–CVD–1100 and its electrochemical oxygen reduction performance [127]. Copyright 2023, American Chemical Society

In the context of the ORR, the catalytic performance of Mn–N–C materials is often limited by the low-spin state of the manganese center and the overly strong adsorption of key reaction intermediates. Moreover, the ambiguous structure–activity relationship between ligand configuration and the true active site poses a fundamental challenge to rational catalyst design. To address these issues, researchers have developed strategies for precise electronic modulation of the Mn active center. Kim et al. systematically investigated the influence of nitrogen ligand types on the electronic structure and ORR activity of Mn single-atom sites (Fig. 7a–d) [125]. Using aerosol spraying combined with ammonia-assisted pyrolysis, they constructed two distinct Mn–N_4_ configurations on crumpled graphene carriers—one with pyrrole-type and the other with pyridine-type nitrogen coordination. Magnetic susceptibility tests and charge density difference analyses revealed that the pyrrole-type N coordination promotes a high-spin state in Mn, which facilitates intermediate desorption and enhances ORR activity, whereas the pyridine-type environment tends to passivate Mn sites. In situ infrared spectroscopy further verified that high-spin Mn serves as the active center for the reaction. In a separate study, Song’s group introduced yttrium (Y) to prepare a MnY/NC catalyst containing both Mn clusters and unsaturated Mn–N_3_ coordination motifs (Fig. 7e, f) [126]. It was found that Y preferentially binds oxygen during high-temperature carbonization, inhibiting Mn oxidation and promoting the formation of an unsaturated coordination structure. This “sacrificial protection mechanism” and the resulting “remote synergistic effect” effectively modulate the electronic state of Mn, significantly improving ORR performance. Additionally, Stracensky et al. adopted a chemical vapor deposition (CVD) approach to avoid the generation of MnOₓ intermediates commonly encountered in conventional synthesis routes (Fig. 7g) [127]. By enabling direct reaction between Mn precursors and N-doped carbon substrates, the CVD method allows in situ formation of a stable Mn–N_4_ structure, thereby enhancing both the structural integrity of the active sites and the efficiency of the ORR pathway.

**Fig. 8:**
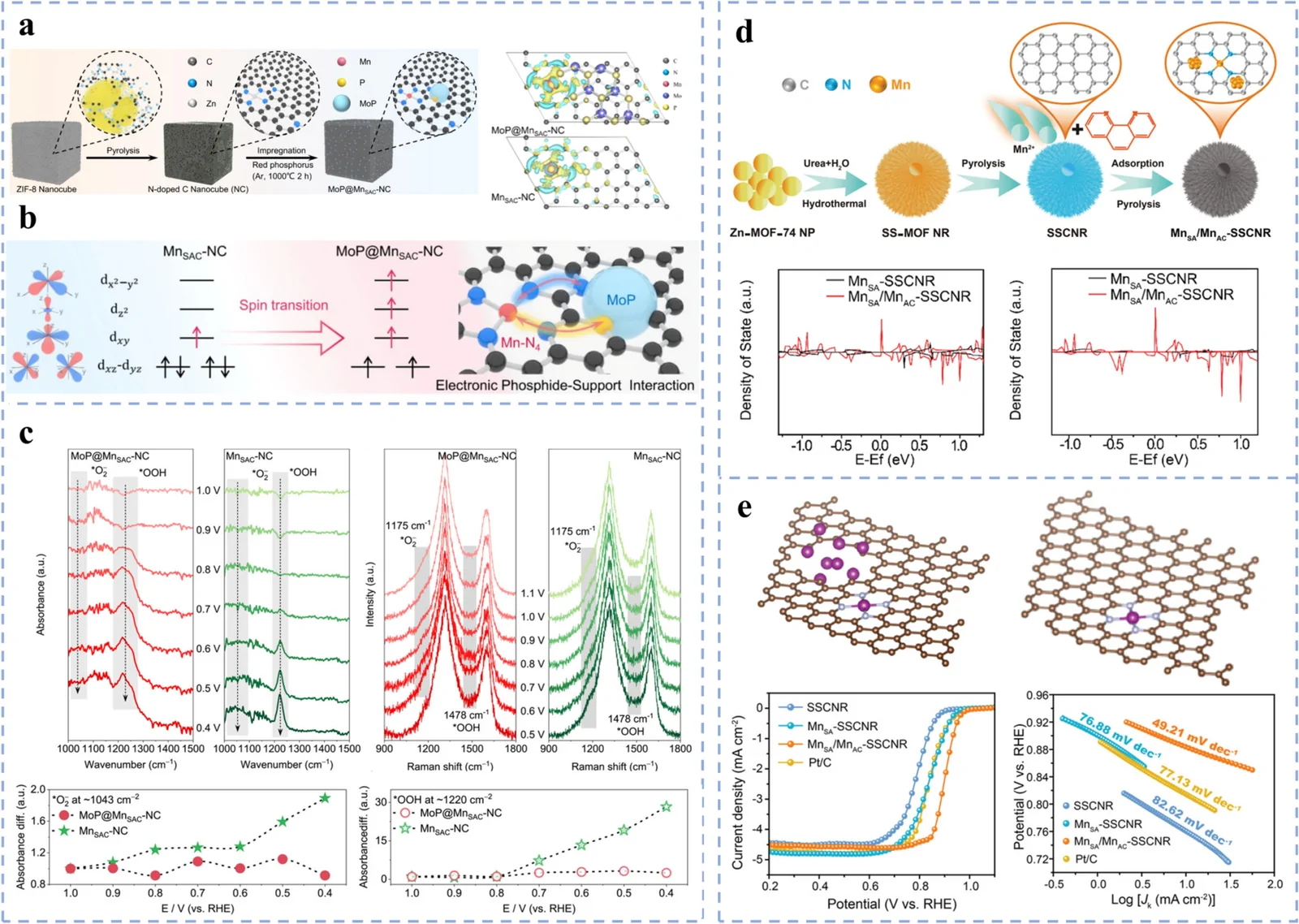
**a** Preparation process of MoP@MnSAC–NC catalyst and its charge density difference distribution. **b** Mechanism of Mn atomic d orbital spin state transition induced by catalyst. **c** In situ FTIR and Raman spectra of MoP@MnSAC–NC and MnSAC–NC at different potentials [128]. Copyright 2025, Wiley–VCH **d** Synthesis process of MnSA/MnAC–SSCNR and its electrocatalytic test. **e** Calculation model of MnSA–SSCNR and MnSA/MnAC–SSCNR and their total density of states (TDOS) and local density of states (PDOS) [129]. Copyright 2024, Wiley–VCH

Based on the previously discussed advances in ligand regulation and electronic structure engineering, research on Mn-based catalysts has expanded to structural-level modifications and interface coupling strategies, enabling more precise control over the Mn center’s spin state and reaction pathway. Luo et al. [128] demonstrated a synergistic enhancement in spin state regulation and intermediate adsorption through the construction of a Mn-based heterostructure with strong interfacial interactions (Fig. 8a–c). Using ZIF-8 as a precursor, the team incorporated Mn and Mo precursors along with red phosphorus through a pyrolysis process to form a composite catalytic material. In this configuration, MoP nanocrystals induce an electronic rearrangement with Mn–N_4_ sites, driving the transition of Mn centers from low-spin to high-spin states. This electronic modulation significantly enhances O_2_ adsorption and activation capabilities. In situ spectroscopic analysis further revealed that this heterostructure exhibits stronger adsorption capacity and accelerated reaction kinetics for O_2_^−^ and OOH* intermediates across multiple working potentials, leading to substantially improved ORR performance. In a complementary approach, Li et al. [129] developed a three-dimensional sea urchin-like hollow structure containing both Mn single atoms and sub-nanoclusters (Fig. 8d, e). This unique architecture enables effective electronic structure modulation through synergistic interactions between the atomic and cluster sites. The incorporation of sub-nanoclusters significantly enhance the electronic state density of Mn–N_4_ sites near the Fermi level, thereby facilitating O_2_ adsorption, promoting O–O bond cleavage, and substantially reducing the reaction energy barrier for the ORR.

Manganese-based catalysts still face several critical challenges, including poor stability in acidic media, incomplete understanding of the synergistic mechanisms between single atoms and clusters, limitations in real-time in situ characterization of electronic structure evolution, and insufficient quantitative studies on multifactor coupling mechanisms [130]. To advance Mn-based ORR catalysts, future research should focus on elucidating the intricate coupling relationships among atomic structure, electronic dynamics, and reaction pathways. This requires precise regulation of manganese active sites through atomic-level control of coordination environments and electronic states, combined with enhanced integration of in situ characterization and theoretical simulations to reveal dynamic structural evolution and catalytic mechanisms during operation [131]. The development of multimetal synergistic systems—such as composite structures incorporating manganese with other 3 d transition metals or rare earth elements—represents a promising direction for enhancing ORR performance. Through these strategies, manganese-based catalysts are expected to evolve from merely demonstrating high activity to achieving balanced performance in stability and practical applicability, thereby establishing a foundation for a new generation of environmentally friendly and efficient ORR catalytic systems [132].

### Ni-Based Catalysts

Ni-based catalysts demonstrate outstanding catalytic activity and pathway selectivity in the ORR, stemming from their distinctive electronic structure, tunable coordination environment, and favorable oxygen affinity [133]. Their structural diversity further provides significant advantages in enhancing reaction selectivity, reducing energy barriers, and improving catalytic durability, making them a key candidate for high-performance ORR electrocatalysts. Recent research has developed a range of effective strategies to achieve precise control over interfacial electron distribution and band structure. These approaches include constructing heterojunction interfaces to promote O_2_ dissociation and OH* desorption, introducing high-entropy atoms to induce strong electric dipole transitions and orbital hybridization, and designing low-valence nickel sites to enhance reaction activity and electron transfer efficiency [134]. The combined application of these strategies has consistently driven performance breakthroughs in Ni-based ORR catalysts, paving the way for their practical implementation in advanced energy conversion devices [135, 136].

**Fig. 9:**
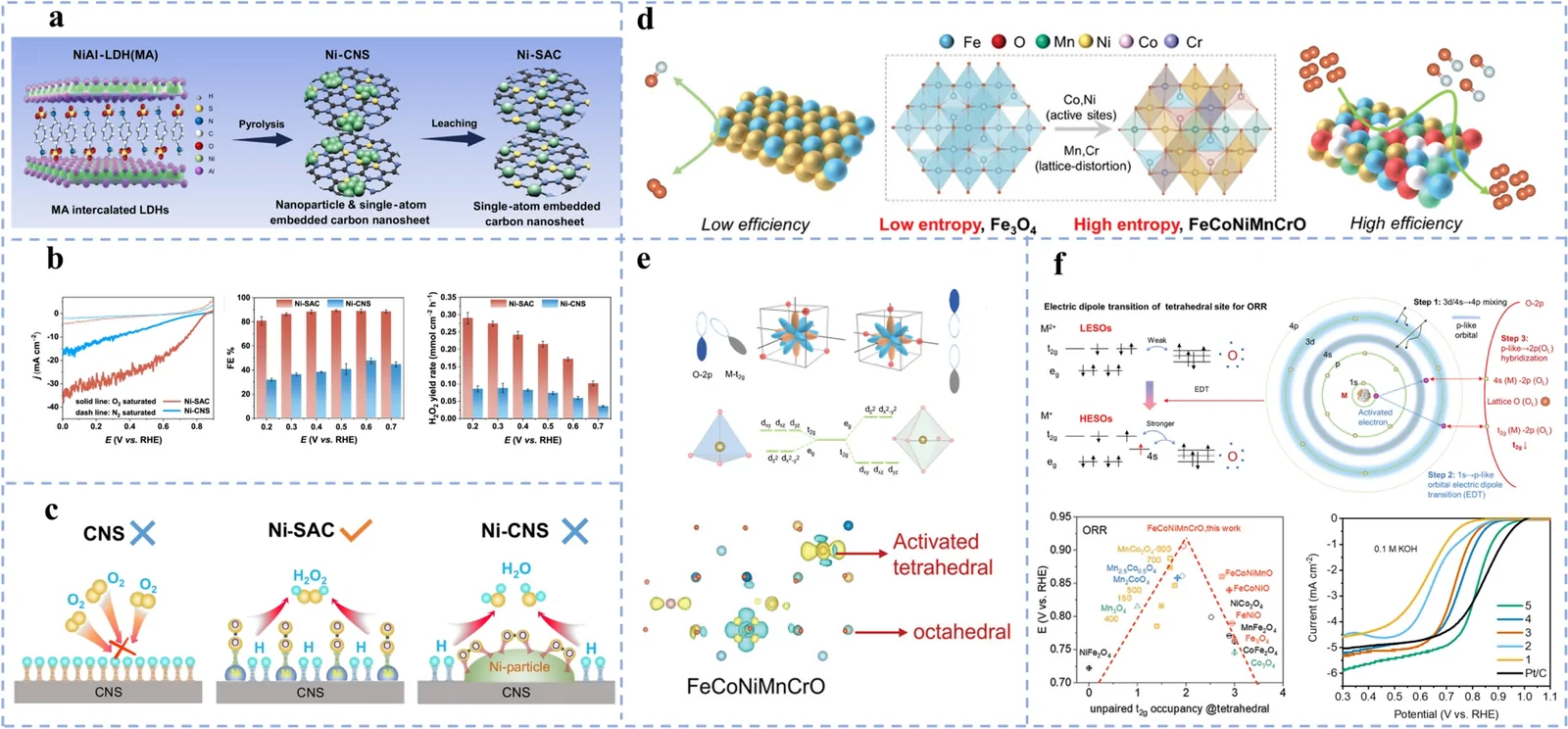
**a** Flowchart of the preparation of Ni–SAC. **b** LSV curves, Faradaic efficiency (FE) and H_2_O_2_ yield of Ni–SAC and Ni–CNS at different potentials. **c** Reaction mechanism of Ni–SAC and Ni–CNS [137]. Copyright 2024, Springer Nature. **d** Characteristics of low-entropy spinel oxides with integrated single octahedral sites and high-entropy spinel oxides with bipolar dual-active sites. **e** Electronic structures of tetrahedron and octahedron in LESO. Differential charge density diagram of Fe_3_O_4_ and FeCoNiMnCrO, density of states projection. **f** High-entropy-induced robust electric dipole transitions enhance ORR activity on tetrahedral sites, volcano diagram of zircon oxides [138]. Copyright 2024, Wiley–VCH

Sun et al. [137] successfully synthesized a Ni-doped carbon nanosheet (Ni–CNS) material containing both Ni nanoparticles and single-atom Ni sites through pyrolysis and acid washing of MA-intercalated NiAl-LDH precursors (Fig. 9a). By selectively removing the particulate Ni species, they further obtained a purely single-atom Ni catalyst (Ni–SAC). A systematic electrochemical comparison among the three materials (Fig. 9b) revealed that Ni–SAC exhibited the highest onset potential, current density, Faradaic efficiency, and H_2_O_2_ yield under oxygen-rich alkaline conditions, demonstrating superior 2e^−^ ORR activity and selectivity over both Ni–CNS and the bare carbon nanosheet (CNS). Further mechanistic analysis (Fig. 9c) indicated that the single-atom Ni sites in Ni–SAC promote selective O_2_ adsorption, while the CNS framework facilitates the generation of active hydrogen species (H*). The synergy between these components significantly enhances H_2_O_2_ production efficiency. In contrast, the presence of Ni nanoparticles in Ni–CNS favors O–O bond cleavage, leading to different reaction selectivity. These results collectively highlight the critical role of atomically dispersed Ni in modulating the ORR pathway for selective H_2_O_2_ synthesis.

In a separate study, Zhang’s group proposed a high-entropy-induced electric dipole transition strategy to construct a spinel oxide (FeCoNiMnCrO) with bipolar dual-active sites for multiscale regulation of the ORR pathway [138]. As illustrated in Fig. 9d, the incorporation of multiple metal elements in FeCoNiMnCrO creates a highly distorted lattice, where Co and Ni serve as the main active centers, while Mn and Cr enhance lattice entropy and structural distortion. This high-entropy configuration significantly outperforms low-entropy Fe_3_O_4_ in bifunctional ORR/OER activity. The study further elucidated the electronic structure evolution at octahedral and tetrahedral sites (Fig. 9e). The high-entropy-induced lattice distortion activates originally inert tetrahedral sites, turning them into novel ORR active centers. Moreover, the electric dipole transition promoted by the high-entropy environment enhances the hybridization between the Co t_2g_ and O 2*p* orbitals (Fig. 9f), resulting in the formation of low-valence tetrahedral Co and high-valence octahedral Ni. This work identifies the “unpaired t_2g_ electron number” as a key electronic descriptor for ORR activity. Combined experimental and theoretical analyses confirm the effectiveness of the high-entropy strategy in regulating electronic structure and enhancing catalytic performance.

**Fig. 10:**
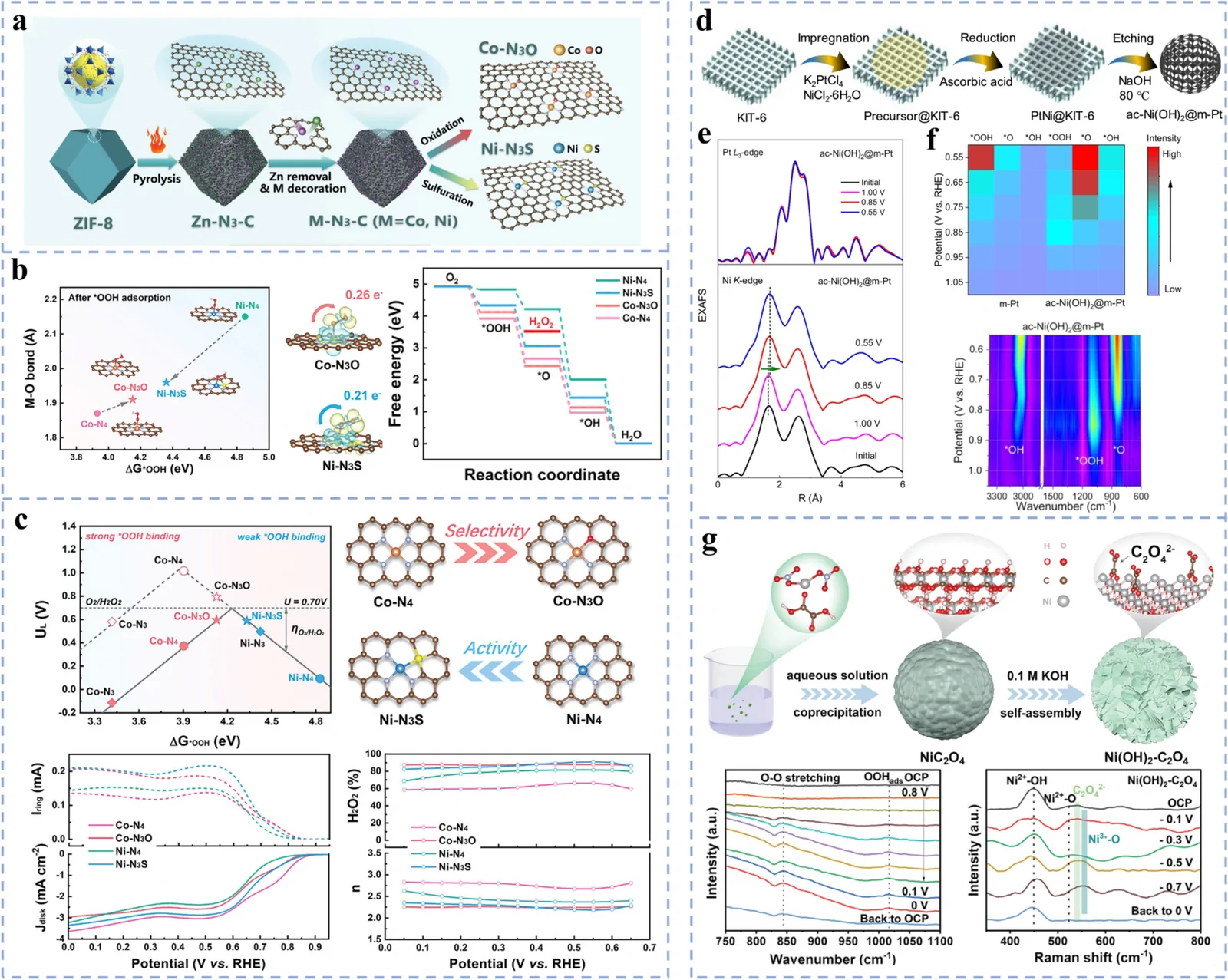
**a** Synthesis process of Co–N_3_O and Ni–N_3_S. **b** Change of metal–oxygen bond length and charge difference density after OOH* adsorption and catalyst ORR free energy path. **c** Volcano plot of potential and free energy, RRDE experimental curve and H_2_O_2_ selectivity and electron transfer number [139]. Copyright 2024, Wiley–VCH. **d** Schematic diagram of the synthesis of the catalyst. **e** Changes in the in situ EXAFS spectrum at different potentials. **f** Differences in the adsorption strength of key intermediates between ac–Ni(OH)_2_@m–Pt and m–Pt in the ORR process [140]. Copyright 2025, Springer Nature. **g** Synthesis path of NiC_2_O_4_ nanoparticles and Ni(OH)_2_–C_2_O_4_ nanoflowers and their in situ spectral characterization in ORR [141]. Copyright 2024, Wiley–VCH

The Pan team developed an electrocatalytic system based on Ni–N_3_S single-atom sites, achieving efficient electrochemical synthesis of H_2_O_2_ through precise electronic structure modulation via first coordination sphere engineering (Fig. 10a) [139]. The adsorption strength of the key OOH* intermediate was found to positively correlate with the metal–oxygen bond length across different coordination structures (Fig. 10b). Both Co–N_3_O and Ni–N_3_S configurations fall within the optimal adsorption range, characterized by moderate binding capacity and low reaction free energy barriers, theoretically favoring selective H_2_O_2_ generation. While Co–N_3_O resides in the strong adsorption region of the ΔG_OOH*_ diagram (Fig. 10c) with higher selectivity, Ni–N_3_S positioned on the weak adsorption side demonstrates superior catalytic activity. Both coordination environments enable high current density and H_2_O_2_ selectivity over a wide potential range, significantly outperforming conventional M–N_4_ structures. The introduction of heteroatoms with varying electronegativities into the first coordination shell effectively tunes the electronic structure of metal centers, enabling precise control over catalytic performance. Liu’s group further demonstrated dynamic manipulation of Pt-based catalyst electronic states through construction of a heterogeneous a–c interface structure (Fig. 10d) [140]. In situ X-ray absorption fine structure (XAFS) analysis (Fig. 10e) revealed potential-dependent electron redistribution between Pt and Ni sites, where Ni initially accepts electrons to activate Pt sites before transferring electrons back to Pt, thereby accelerating oxygen protonation and enhancing reaction kinetics. Synchrotron radiation experiments (Fig. 10f) confirmed that this electron redistribution promotes OH* intermediate formation, facilitating the efficient 4e^−^ ORR pathway. The interfacial structure between a and c phases achieves synergistic enhancement of both activity and stability through electronic state modulation and intermediate adsorption behavior regulation. Chen’s group elucidated the synthesis process of Ni(OH)_2_–C_2_O_4_ electrocatalysts and their structural evolution during the 2e^−^ ORR process (Fig. 10g) [141]. In alkaline environments, NiC_2_O_4_ spontaneously transforms into Ni(OH)_2_–C_2_O_4_, where oxalate self-assembles and adsorbs onto the Ni(OH)_2_ surface, forming a stable functional layered structure. In situ infrared spectroscopy revealed enhanced O–O stretching vibrations and intensified OOH* adsorption signals with potential variation, indicating that oxalate modification promotes the adsorption and activation of key intermediates. In situ Raman spectroscopy demonstrated reversible formation and disappearance of NiOOH characteristic peaks during the ORR process, confirming the remarkable reversibility and stability of nickel active sites. The persistent oxalate signals throughout the reaction suggest its structural integrity, indicating that oxalate not only stabilizes the overall structure but also plays a crucial role in modulating the electronic structure to facilitate intermediate adsorption/desorption and enhance electrocatalytic activity.

Despite their potential in the ORR, Ni-based catalysts often exhibit limited electrocatalytic activity and face challenges in forming efficient active centers. These materials typically suffer from insufficient electrical conductivity and structural vulnerability, particularly in both alkaline and acidic environments where nickel species are prone to dissolution or migration, leading to progressive degradation of catalytic integrity and rapid activity decay [142]. Moreover, TMCs based on nickel generally demonstrate restricted capability for O_2_ adsorption and activation, along with inadequate control over key intermediates such as OOH*, which collectively impair ORR pathway selectivity and electron transfer efficiency [143]. In light of these structural and performance constraints, advanced regulation of Ni-based systems remains a compelling research direction. Future studies should prioritize the rational design of efficient nickel active sites with tunable electronic structures, the development of novel low-valent nickel centers, and the construction of stable composite materials through heterogeneous electrocatalysis strategies. Special emphasis should be placed on enhancing structural durability and dissolution resistance under both strongly basic and acidic conditions to facilitate practical implementation.

### Co-Based Catalysts

Cobalt-based electrocatalysts have emerged as promising candidates for the ORR, owing to their tunable electronic structure, effective intermediate adsorption capability, and cost-efficient characteristics [144–146]. Recent studies indicate that strategic manipulation of local atomic configuration, spin states, and exposed crystal planes can significantly enhance their catalytic performance. However, as research advances toward more precise control of active site structure and electronic environments, Co-based TMCs face two major challenges in ORR applications: conventionally pyrolyzed Co–N–C structures often suffer from poorly controlled coordination geometry, non-uniform charge distribution, and electronic state heterogeneity at active centers, which collectively impede a systematic understanding of their catalytic behavior and spatial dynamics. Moreover, the typical Co–N₄ configuration shows limited ability to balance reaction selectivity and kinetic activity during the adsorption regulation of key intermediates such as OOH*, thereby restricting its practical utility in selective H_2_O_2_ production and the 4e^−^ ORR pathway. In response, researchers have pursued precise coordination design, electronic modulation, and orbital hybridization strategies to overcome the limitations of conventional Co–N_4_ sites. By adjusting the electronegativity of the Co center, local spin configuration, and ligand field strength, they have realized directed ORR pathway control and enhanced catalytic performance [147–149].

**Fig. 11:**
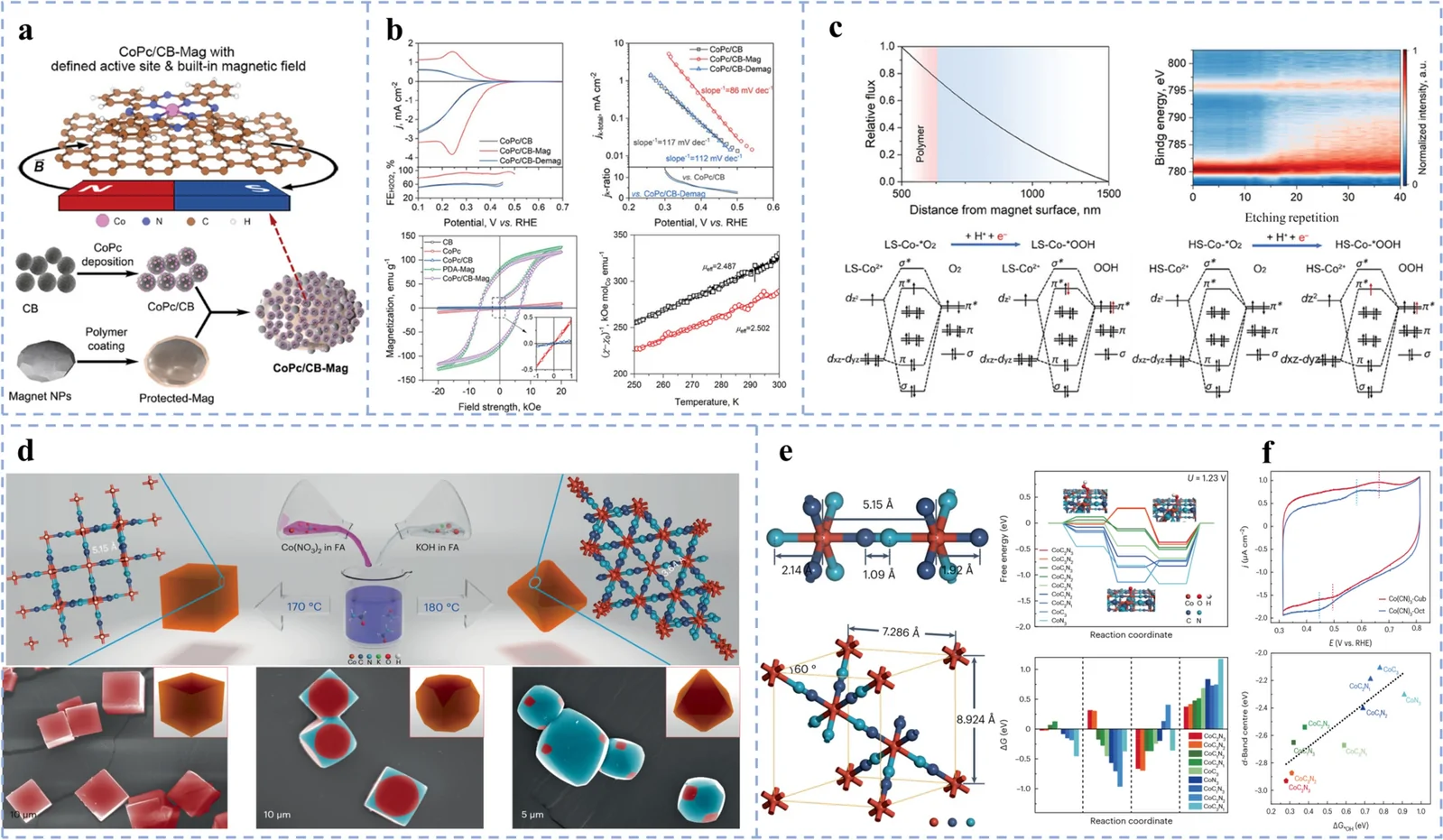
**a** Schematic diagram of the synthesis of CoPc/CB–Mag composites and the catalytic system of active sites and built-in magnetic fields. **b** Performance in ORR as well as magnetic susceptibility and hysteresis loops. **c** Simulation of the effect of the polymer layer on the surface of magnetic particles on the spatial distribution of magnetic flux density and Co 2*p* XPS depth profile and orbital energy level diagram [150]. Copyright 2024, Wiley–VCH. **d** Three types of Co(CN)_3_ microcrystals, namely, cube (Cub), truncated cube and octahedron (Oct), were synthesized. **e** Local atomic structure and unit cell model and free energy diagram of Co(CN)_3_–Cub were revealed. **f** Relationship between the CV curve and the OH* adsorption energy and the *d* band center of the Co site [151]. Copyright 2023, Springer Nature

Yu and colleagues developed a heterogeneous molecular electrocatalyst system featuring an integrated built-in magnetic field, constructing a hierarchical CoPc/CB–Mag catalyst (Fig. 11a) [150]. This catalyst was synthesized by depositing cobalt phthalocyanine onto carbon black and combining it with polydopamine-coated magnetic nanoparticles, forming an interface structure characterized by distinct Co–N_4_ active sites and the synergistic effect of the built-in magnetic field. In the ORR, the CoPc/CB–Mag catalyst demonstrated superior onset potential and current density, along with a lower Tafel slope and charge transfer resistance (Fig. 11b). Its catalytic activity and electron transport performance were significantly enhanced compared to the control group. Subsequent X-ray photoelectron spectroscopy (XPS) analysis, flux simulation, and *d* orbital energy level investigations (Fig. 11c) revealed that the built-in magnetic field induces a transition of the cobalt center from a low-spin to a high-spin state, elevating the d_22_ orbital energy level and thereby enhancing the adsorption and activation capabilities for O_2_ and OOH* intermediates. This synergy effectively improves ORR performance and introduces a novel strategy for modulating the electronic structure of single-atom catalytic sites via magnetic field induction. In a separate study, Sun et al. [151] developed a morphology-controlled Co(CN)_3_ microcrystalline catalyst through structural regulation of the cobalt coordination environment (Fig. 11d). By adjusting reaction temperature and ligand concentration, three distinct crystal morphologies—cube, truncated cube, and octahedron—were successfully synthesized. The systematic exposure of different crystal facets effectively modulated the local coordination structure of cobalt. Among these, the five-coordinate Co–C_2_N_3_ site, together with the corresponding free energy profile (Fig. 11e), exhibited the lowest overpotential and reaction free energy barrier. The Co(CN)_3_–Cub variant demonstrated a higher onset potential and half-wave potential, as well as a lower Tafel slope (Fig. 11f), achieving peak power density of 1.67 W cm^−2^ in fuel cells while maintaining excellent cycling stability.

While Co-based catalysts have demonstrated considerable progress in the ORR, they continue to face critical scientific and engineering challenges that impede their practical implementation, necessitating further fundamental research and technological breakthroughs [152]. Currently, the inability to systematically detect the in situ dynamic behavior of key reaction intermediates (e.g., OOH* and OH*) at operating potentials hinders the precise identification of rate-determining steps and actual active sites. Furthermore, enhancing H_2_O_2_ selectivity often occurs at the expense of intrinsic catalytic activity, making the coordinated regulation of both high activity and high selectivity a central bottleneck [153]. Additionally, the long-term stability and environmental adaptability of these TMCs in practical devices—particularly under harsh conditions such as strongly acidic or basic electrolytes—require significant improvement. Future research should prioritize the design of novel coordination structures and the development of advanced electronic state control strategies, including interface polarization, spin state manipulation, and local strain engineering. The integration of in situ and quasi-in situ multiscale characterization techniques with first-principles simulations will be essential to elucidate the underlying catalytic mechanisms. Such integrated efforts are expected to accelerate the practical application of cobalt-based heterogeneous electrocatalysis systems in sustainable energy conversion and green chemical synthesis.

### Cu-Based Catalysts

While Cu-based catalysts have demonstrated significant potential in electrochemical applications—particularly in the carbon dioxide reduction reaction where they exhibit notable electrocatalytic performance their role in the ORR is also attracting growing interest [154–157]. Although platinum-based catalysts generally remain more active for the ORR, Cu-based catalysts are increasingly regarded as promising alternative materials for energy conversion systems such as fuel cells and metal–air batteries, owing to their cost-effectiveness, natural abundance, and considerable stability [158, 159].

**Fig. 12:**
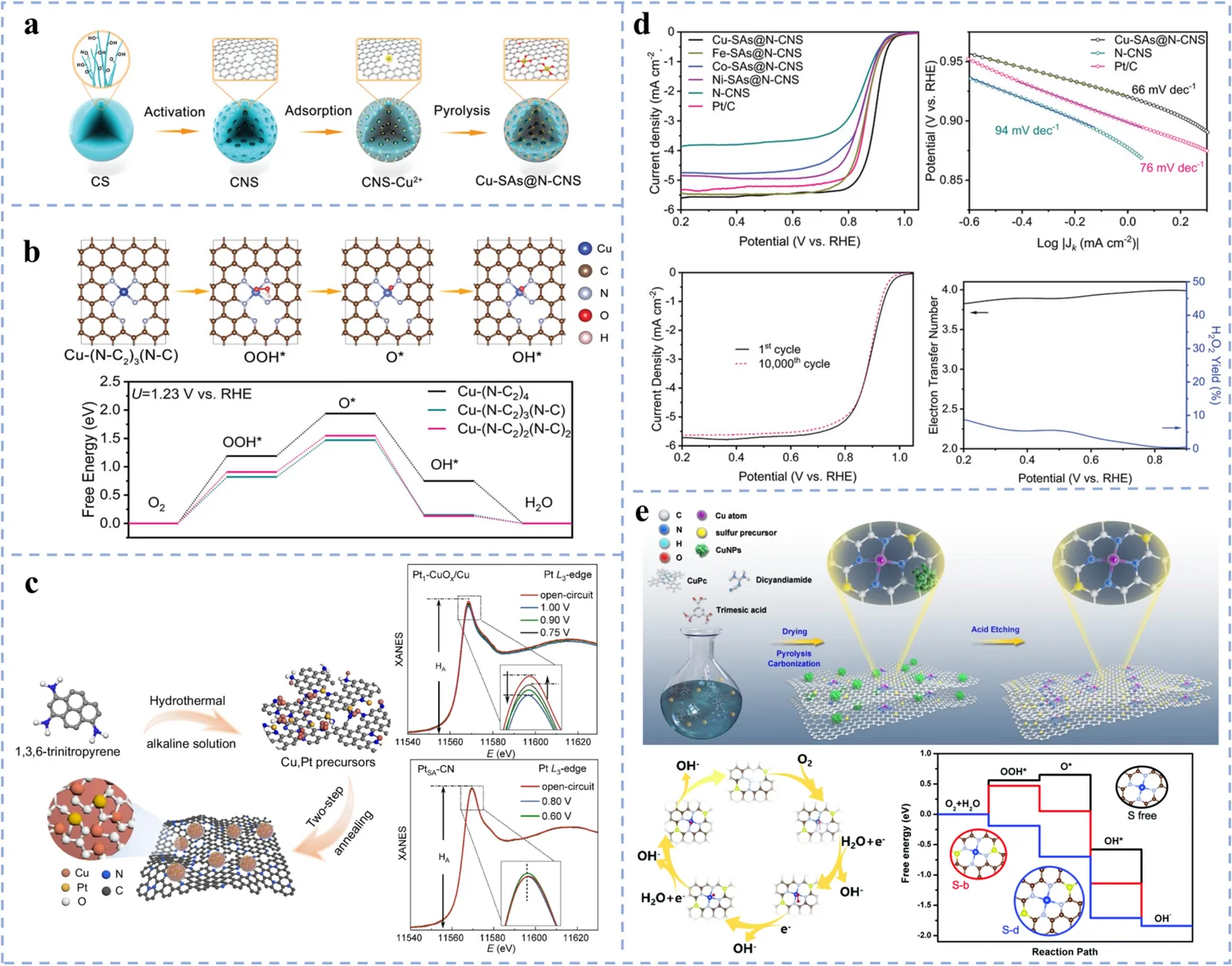
**a** Schematic diagram of the synthesis process of Cu–SAs@N–CNS. **b** Adsorption configurations of intermediates (OOH*, O*, OH*) and free energy diagrams of 4e^−^ ORR paths in three different coordination environments [160]. Copyright 2021, Wiley–VCH. **c** Synthesis process of Pt_1_–CuOx/Cu single-atom catalyst and its in situ structural evolution and in situ XAFS characterization during electrochemical reaction, as well as XANES of comparative samples [161]. Copyright 2024, Springer Nature. **d** Electrocatalytic performance of various transition metal single-atom catalysts in ORR as well as H_2_O_2_ yield and electron transfer number. **e** Synthesis process of sulfur-modified copper single-atom catalyst and its theoretical study in ORR [158]. Copyright 2019, Royal Society of Chemistry

SACs represent a fundamental strategy for achieving efficient metal atom anchoring and precise regulation of active sites, owing to their atomic-level dispersion and structural tunability. The development of Cu–SACs holds particular significance for understanding structure–performance relationships in ORR catalysts. To systematically investigate these relationships, the Zong team engineered a structurally defined Cu–SAC with tailored defects and comprehensively studied its ORR activity (Fig. 12a) [160]. Their analysis of intermediate adsorption behavior and reaction pathways across various Cu–N_4_ coordination structures revealed that the Cu–(N–C_2_)_3_(N–C) configuration exhibited the lowest OOH* adsorption free energy (Fig. 12b), indicating superior reaction kinetics and intrinsic activity. These findings underscore the critical role of defect engineering in modulating electronic structure and enhancing the catalytic performance of single-atom systems. In a complementary approach, Zhou et al. [161] developed an efficient ORR catalyst based on copper nanocomposites (Fig. 12c), constructing a CuO_x_/Cu nanostructure with tunable valence states. The combination of Cu^2+^ and zero-valent copper created a versatile valence electron reservoir that enabled dynamic regulation of the electronic state of supported single-atom platinum, ultimately optimizing catalytic performance. This strategy demonstrated excellent 4e^−^ ORR selectivity and remarkable electrocatalytic stability (Fig. 12d). Addressing key challenges such as SAC instability, unmodifiable electronic structures, and inefficient intermediate adsorption, Jiang et al. [158] developed a single-atom copper catalyst leveraging atomic interface effects for enhanced ORR performance (Fig. 12e). Through a strategy combining acid etching with sulfur–nitrogen synergistic doping, they effectively modulated the electronic structure and coordination environment of Cu single atoms. This approach maintained copper in a low-valence state, optimizing the adsorption and dissociation of key intermediates (OOH* and O*), thereby significantly improving overall ORR performance.

Through the implementation of defect engineering, valence state regulation, and interface doping strategies, researchers have successfully developed a series of structurally optimized Cu–SACs. These advances have enabled effective optimization of electronic structures and intermediate adsorption behaviors, addressing key limitations of Cu-based catalysts such as insufficient activity, inflexible electronic states, and poor stability. However, copper active sites typically exist in + 1 or + 2 valence states, where the limited number of *d* orbital electron impedes efficient O–O bond cleavage, consequently favoring the 2e^−^ pathway for H_2_O_2_ formation [162]. Furthermore, single-atom copper systems exhibit constrained electron transfer capability and reaction kinetics due to their rigid valence states and challenging coordination environments, making them susceptible to migration or dissolution under electrochemical conditions, which compromises active site stability. Future research on Cu-based ORR catalysts should prioritize several key directions: dynamic modulation of the copper center’s electronic structure; in situ analysis of valence state evolution and coordination environment reconstruction; and mechanistic investigation of the copper site’s behavior throughout the ORR cycle. Additional efforts should focus on constructing interface structures with electronic regulation capability to enable reversible electronic coupling at copper centers, enhancing active site stability and accessibility through defect engineering and hierarchical pore structure design. The integration of first-principles calculations with in situ spectroscopic techniques will be crucial for identifying rate-determining steps in intermediate adsorption and reaction pathways, thereby establishing a theoretical foundation for the rational design of high-performance Cu-based ORR catalysts.

### Other Transition Metal Catalysts

Beyond the transition metals previously discussed, rare earth materials demonstrate distinctive advantages for electrocatalytic applications due to their unique electronic configurations, tunable valence states, and flexible coordination characteristics. These properties enable precise regulation of reaction intermediate adsorption strength, optimization of electron and proton transfer pathways, and enhanced stabilization of active site structures. Such capabilities make rare earth materials particularly promising for accelerating reaction kinetics, improving catalytic selectivity, and ensuring electrochemical stability—especially under demanding operational conditions requiring long-term durability. The unique 4*f* orbital configuration and multielectron characteristics of rare earth elements provide exceptional electronic regulation capabilities in the ORR. Through *f–p–d* orbital hybridization or asymmetric coordination with ligands such as oxygen, nitrogen, and chlorine, these materials can effectively modulate the electronic structure of transition metal active centers. This electronic optimization improves intermediate adsorption behavior, reduces reaction energy barriers, and accelerates overall reaction kinetics. Simultaneously, certain non-rare earth metal materials contribute significantly to enhancing catalytic performance by inhibiting competing reaction pathways and extending catalyst service life. Their exceptional chemical stability, resistance to oxidative damage, and structural controllability make them valuable complementary components in the development of high-performance, durable ORR catalysts. The synergistic integration of rare earth elements with these stable non-rare earth materials represent a promising direction for advancing next-generation electrocatalytic systems with both enhanced activity and prolonged operational lifetime [163–165].

**Fig. 13:**
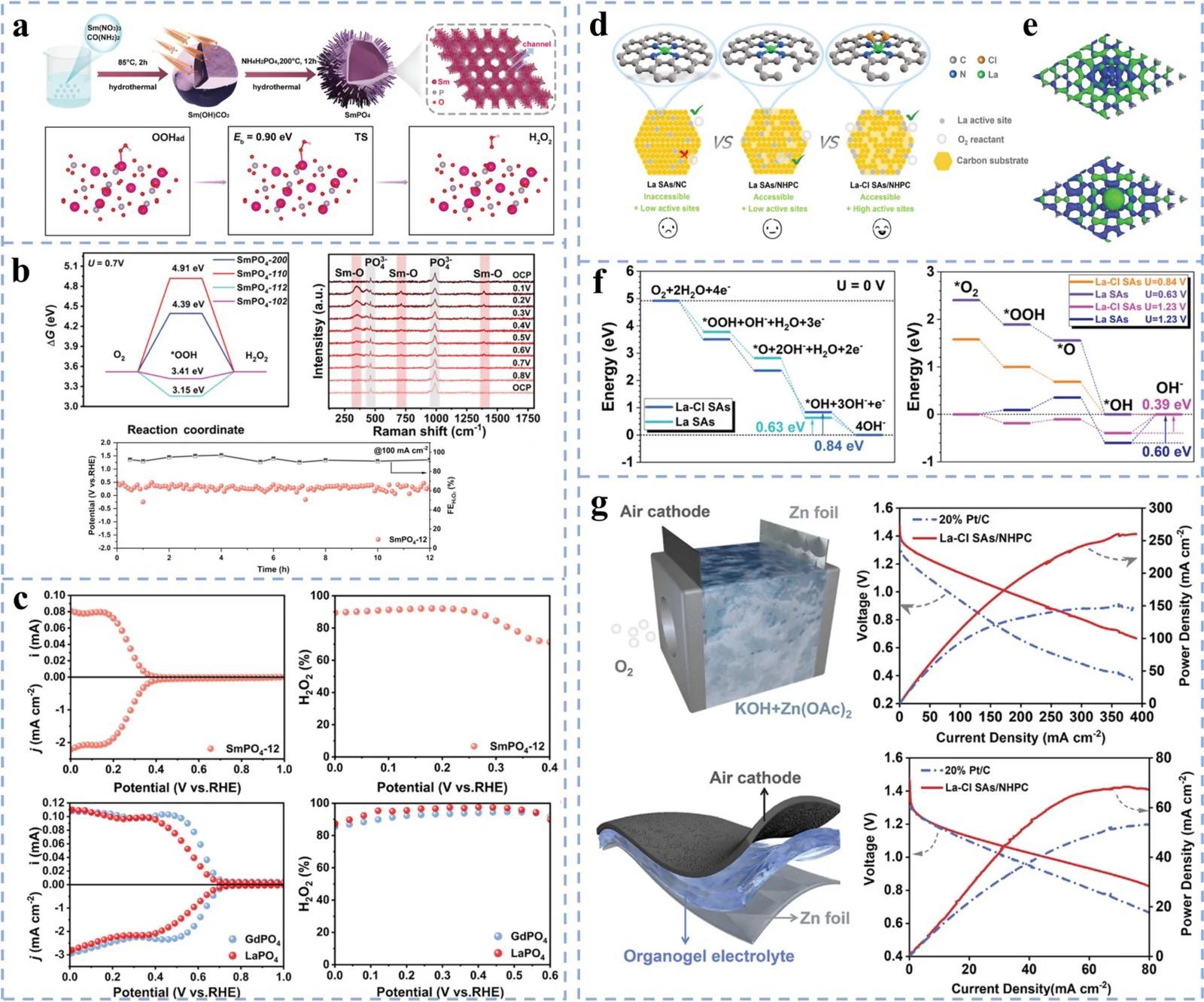
**a** Schematic diagram of the synthesis of SmPO_4_ nanospheres and the atomic model of the initial state, transition state and final state of the OOH* to H_2_O_2_ path. **b** Different crystal planes have different reaction barriers for the 2e^−^ ORR path at U = 0.70 V and in situ Raman spectra and stability tests under gas diffusion electrode configuration [163]. Copyright 2025, Wiley–VCH. **c** LSV curve of catalyst and H_2_O_2_ yield. **d** Schematic diagram of the structures of three different configurations of La single-atom catalysts. **e** Comparison of the electron orbital distribution of La–Cl SAs and La SAs near the Fermi level: blue is the bonding orbital, green is the antibonding orbital, **f** ORR free energy path of La–Cl SAs and La SAs at U = 0 V and different applied potentials. **g** Actual performance of La–Cl SAs/NHPC in aqueous and flexible zinc–air batteries is introduced [164]. Copyright 2025, Wiley–VCH

**Fig. 14:**
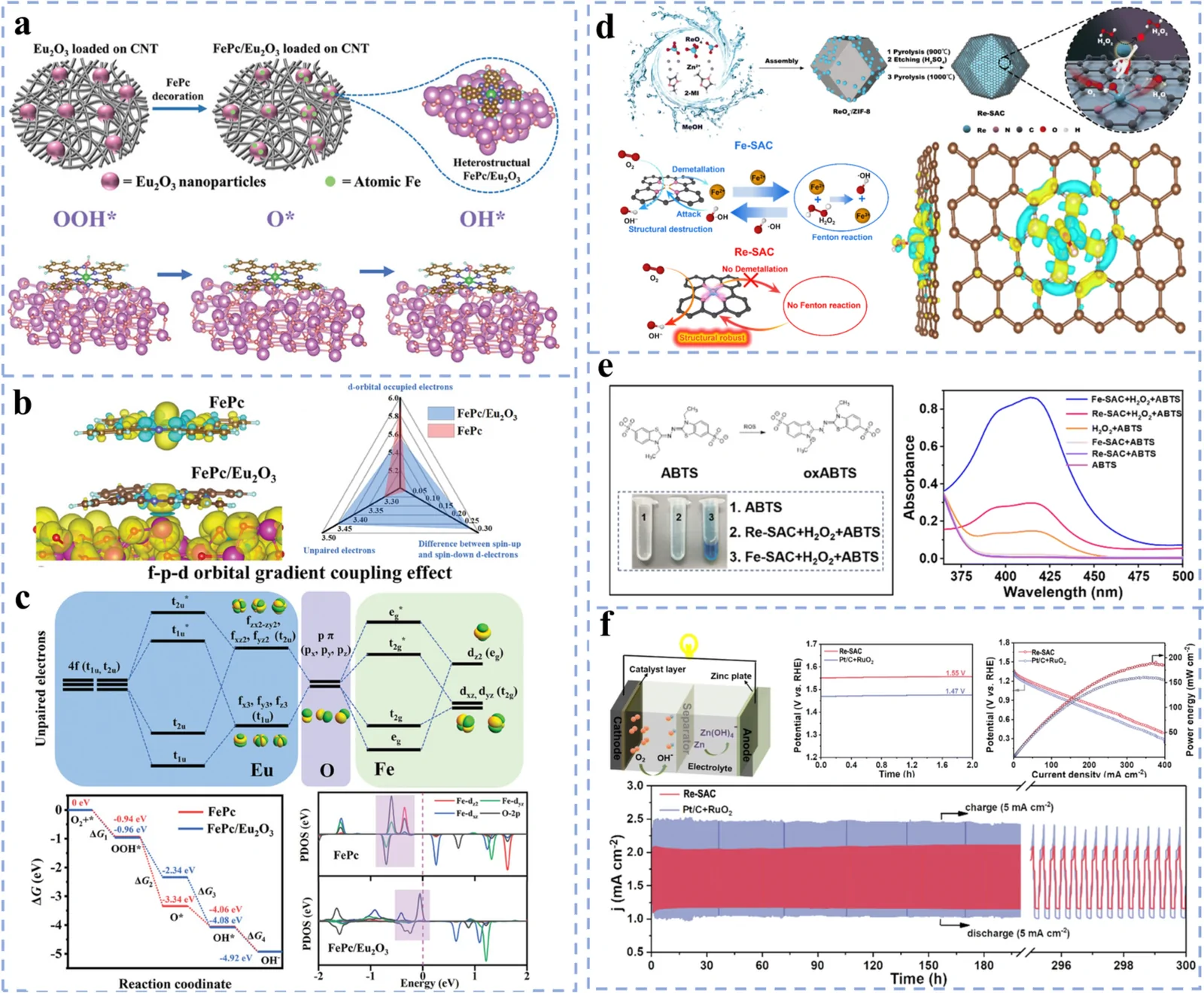
**a** Synthesis path of FePc/Eu_2_O_3_/CNT and the optimized configuration after OOH*, O*, and OH* adsorption. **b** Comparison of the electron spin density distribution of FePc and FePc/Eu_2_O_3_ and the degenerate diagram of the d orbital occupation of Fe atoms. **c** Comparison of the *f–p–d* orbital gradient coupling effect and free energy diagram as well as the density of states after OOH* adsorption [165]. Copyright 2025, Wiley–VCH. **d** Synthesis route of Re–SAC and its inherent anti-Fenton reaction characteristics and the differential charge density diagram showing the redistribution of electrons in Re–SAC (light yellow for charge accumulation and cyan for charge depletion). **e** Schematic diagram of the reaction of ROS with ABTS and the color map and UV–Vis absorption spectrum of the solution after the reaction. **f** Schematic diagram of the zinc–air battery device and performance test results [166]. Copyright 2024, Royal Society of Chemistry

In recent years, rare earth-based catalysts have attracted considerable research attention owing to their distinctive structural characteristics and unique electronic properties. Liu et al. reported an efficient two-electron ORR catalyst utilizing rare earth phosphate materials (Fig. 13a) [163]. Their study systematically investigated the intrinsic mechanism of H_2_O_2_ production, with particular focus on the adsorption and dissociation behavior of the key OOH* intermediate. By integrating theoretical calculations with in situ Raman spectroscopy, they elucidated the variation in OOH* adsorption free energy across different crystal planes (Fig. 13b). The Sm site was identified as the primary active center, exhibiting exceptional capability for intermediate adsorption and release. Electrochemical evaluation using a rotating ring disk electrode (RRDE) demonstrated outstanding 2e^−^ ORR performance (Fig. 13c), achieving over 93% H_2_O_2_ selectivity with remarkable stability under alkaline conditions. The layered SmPO_4_ hollow structure, characterized by open channels, optimal metal spacing, and abundant proton transport pathways, contributes to its superior catalytic activity, selectivity, and structural stability. In a separate study, Yin et al. synthesized a La–SAC through chlorine axial coordination activation and systematically investigated its electronic structure regulation and catalytic mechanism [164]. Compared to conventional La–SACs, this modified catalyst exhibits a lower reaction energy barrier and increased density of exposed active sites. Figure 13d, e illustrates the distribution and coordination structure of La atoms within the carbon substrate. The energy barrier for the key intermediate transition from OOH* to OH* was merely 0.39 eV (Fig. 13f), significantly lower than that of conventional La–SACs, indicating substantially enhanced ORR kinetics. When applied as an air cathode in both liquid and flexible solid-state zinc–air batteries, this catalyst demonstrated superior discharge voltage, power density, and flexibility, outperforming the 20% Pt/C control group **(**Fig. 13g**)**. Furthermore, Cheng et al. developed a FePc/Eu_2_O_3_ composite catalyst based on f–p–d (Eu–O–Fe) gradient orbital coupling (Fig. 14a) [165]. Through the construction of a heterostructure comprising FePc and Eu_2_O_3_, atomic Fe site modification was successfully achieved on the carrier. The Eu–O–Fe bridging structure significantly enhances the adsorption behavior of critical intermediates (OOH*, O*, and OH*), thereby reducing the reaction energy barrier. Differential charge density and electronic state distribution analyses (Fig. 14b) revealed that electron redistribution within the FePc/Eu_2_O_3_ system facilitates improved electron transfer. Quantitative comparison of *d* orbital electronic states and unpaired electron numbers between this system and FePc provided detailed insight into the *f–p–d* gradient orbital coupling mechanism. The coupling of the Eu f orbital with the O *p* orbital stimulates *d* orbital transitions in Fe, increasing e_g_ orbital occupancy and transforming the Fe center from a low-spin to a medium-spin state. This electronic reorganization enhances the cooperative adsorption and desorption capabilities for reaction intermediates. Additionally, the incorporated f band functions as an electron buffer layer, effectively mitigating electron loss and maintaining structural stability (Fig. 14c). These studies collectively demonstrate that rare earth elements, through their unique 4*f* electronic configurations and flexible coordination characteristics, provide powerful strategies for designing advanced ORR catalysts with tailored activity, selectivity, and stability.

During the investigation, researchers have discovered that various TMCs demonstrate significant anti-Fenton reaction characteristics. Yue et al. [166] systematically studied a Re–SAC, thoroughly examining its exceptional anti-Fenton performance and underlying mechanism. The Re–SAC effectively suppresses the formation of OH* radicals in the presence of H_2_O_2_, which is attributed to its stable Re–N_4_ coordination structure that prevents oxidative degradation of the carbon support. Analysis of charge density distribution and electronic structure revealed pronounced electron localization and enhanced oxidative resistance at the Re active sites (Fig. 14d). Subsequent Fenton reaction tests using ABTS probes (Fig. 14e) confirmed the remarkable anti-Fenton capability of the Re–SAC system. When implemented in zinc–air batteries (Fig. 14f), the Re–SAC demonstrated outstanding practical performance, including a high open-circuit voltage, substantial maximum power density, and stable charge–discharge cycling over 300 h, surpassing the performance of commercial Pt/C + RuO_2_ benchmarks.

Despite these advantages, the broader adoption of Re–SACs in ORR catalysis remains limited. Compared with Fe–SACs, Re–SACs suffer from higher material cost, lower elemental abundance, and a narrower optimal operating window, which restrict their scalability and general applicability. Moreover, the strong oxidative resistance and moderated *OOH binding at isolated Re sites, while beneficial for suppressing radical-induced degradation, may compromise catalytic activity or pathway flexibility under certain ORR conditions. Consequently, Re–SACs are currently better suited for stability-critical or radical-sensitive applications, rather than serving as a universal replacement for Fe-based single-atom catalysts.

Rare earth-based supports demonstrate exceptional structural stability and dissolution resistance, maintaining remarkable electrochemical durability particularly under alkaline conditions. Future investigations should prioritize elucidating the orbital hybridization mechanisms between rare earth elements and transition metals, developing precise single-atom regulation strategies, and constructing enhanced architectures with optimal coordination in conductive matrices. Concurrently, research should address the optimization of their stability in acidic media to expand their applicability in energy conversion devices such as fuel cells and metal–air batteries. Complementarily, TMCs exhibit significant activity in the ORR due to their tunable *d* orbital electronic structures and variable oxidation states. Future research directions should focus on precise modulation of d band center positioning and intermediate adsorption behavior, along with constructing multimetal synergistic systems or heteroatom coordination environments to enhance catalytic selectivity and electron transfer efficiency. Substantial efforts should also be directed toward developing effective anti-dissolution strategies—including interface engineering and protective carbon layer coating—to address stability challenges in acidic environments. These coordinated advances will establish a robust foundation for practical device applications across diverse energy conversion systems.

## Application of Transition Metal Heterogeneous Catalysts in ORR

Transition metal-based heterogeneous electrocatalysis demonstrates significant potential across diverse applications in the ORR. In energy conversion and storage technologies such as fuel cells and metal–air batteries, these TMCs serve as critical components for enhancing device efficiency and prolonging cycle life, owing to their exceptional catalytic activity, remarkable durability, and cost-effectiveness [167]. Furthermore, through precise regulation of the electronic structure and local coordination environment of the metal centers, these catalysts can achieve highly selective 2e^−^ reduction pathways, enabling efficient synthesis of H_2_O_2_. This capability provides innovative and sustainable solutions for environmental remediation, green chemical production, and medical disinfection. A central challenge in advancing the design and optimization of these catalytic systems lies in the precise control between 2e^−^ and 4e^−^ ORR pathways to meet distinct application requirements [168]. This section systematically reviews recent progress and future prospects of transition metal-based heterogeneous catalysts, with a focus on two key directions: electrocatalytic synthesis of H_2_O_2_ and their implementation in energy devices.

### Metal–Air Batteries

The ORR serves as a fundamental process governing the performance of metal–air batteries, where its catalytic efficiency directly determines key operational parameters including energy density, power output, and cycling stability. Owing to their high theoretical energy density and environmental sustainability, metal–air battery systems represent a primary application domain for advanced ORR catalyst development [169, 170]. In recent years, TMCs and their derived materials have demonstrated remarkable advantages in enhancing ORR kinetics and selectivity. This is attributed to their multivalent characteristics and highly tunable electronic structures, which enable precise regulation of intermediate adsorption/desorption behavior. Consequently, these materials have emerged as a crucial category of electrocatalysts for developing next-generation metal–air batteries with superior performance.

**Fig. 15:**
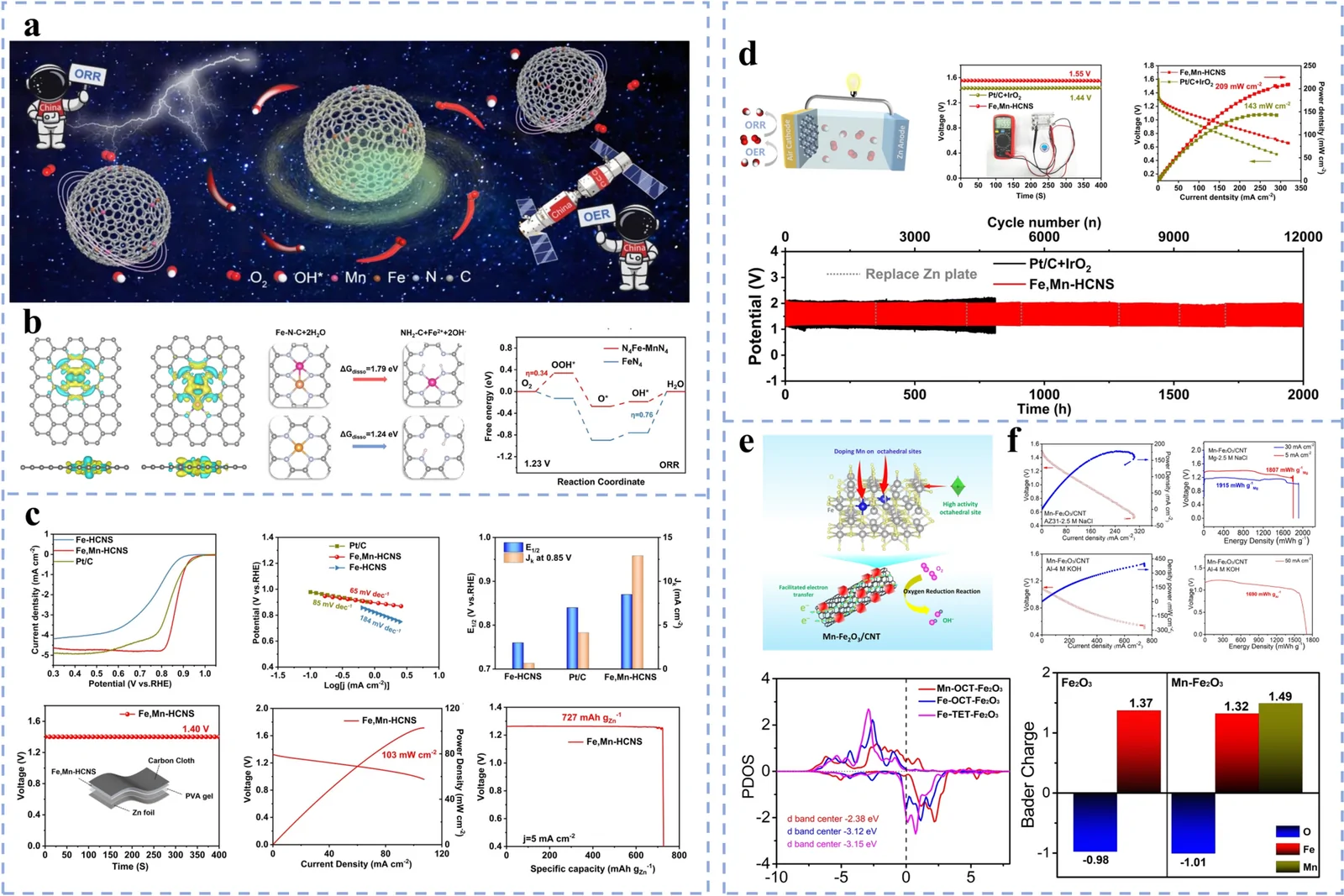
**a** Synergistic mechanism of Fe and Mn promoting ORR and OER respectively in the catalytic cycle. **b** Differential charge density and free energy change of FeN_4_ and Fe–MnN_4_ and state density analysis. **c** Fe–Mn–HCNS electrocatalytic performance test and constructed flexible zinc–air battery [171]. Copyright 2024, Elsevier. **d** Schematic diagram of the battery structure of Fe, Mn–HCNS and the performance of alkaline zinc–air batteries in practical applications. **e** Octahedral sites, state density analysis and Bader charge and charge density difference diagram in Mn-doped activated Fe_2_O_3_. **f** Polarization performance and power density of zinc–air batteries and aluminum–air batteries constructed with Mn–Fe_2_O_3_/CNT [172]. Copyright 2025, Wiley–VCH

Among numerous studies exploring the regulation of Fe-based catalysts for zinc–air batteries, Ji et al. reported a Fe, Mn–HCNS catalyst where Mn atoms modulate the electronic structure of FeN_4_ sites on hollow carbon nanospheres (Fig. 15a) [171]. Their findings demonstrate that Mn incorporation effectively modifies the electronic configuration of FeN_4_ centers (Fig. 15b), reducing reaction free energy barriers and enhancing kinetic processes. Electrochemical characterization revealed that Fe, Mn–HCNS exhibits a more positive half-wave potential and lower Tafel slope in the ORR, ultimately enabling superior performance in practical rechargeable zinc–air battery tests (Fig. 15c). These results comprehensively confirm the crucial role of Mn-induced synergistic regulation in enhancing bifunctional electrocatalytic performance, validating the catalyst’s effectiveness for advanced zinc–air batteries. In a complementary multivalent synergistic strategy, Qiu’s research group engineered a Mn-doped γ-Fe_2_O_3_/CNT composite catalyst (Mn–Fe_2_O_3_/CNT) as high-performance air electrode material for metal–air batteries [172]. Selective incorporation of Mn atoms into octahedral vacancies of the γ-Fe_2_O_3_ lattice enables precise modulation of Fe active sites’ electronic structure. This catalyst demonstrated exceptional discharge performance, high open-circuit voltage, and prolonged operational stability in both Mg–air and Al–air battery systems (Fig. 15d), highlighting its broad applicability in practical devices. Structural modeling and d band density of states analysis (Fig. 15e) reveal that Mn doping not only enhances O_2_ molecule adsorption but also establishes synergistic interactions with Fe sites, effectively regulating the ORR pathway. Subsequent free energy calculations and charge density difference analysis (Fig. 15f) demonstrate that Mn incorporation reduces reaction energy barriers while enhancing OH* intermediate adsorption capacity, significantly improving catalytic reaction kinetics. This integrated theoretical and experimental investigation elucidates the critical role of octahedral Mn doping in enhancing the ORR performance of Fe-based catalysts, providing valuable insights for developing efficient non-precious metal catalysts for metal–air battery applications.

**Fig. 16:**
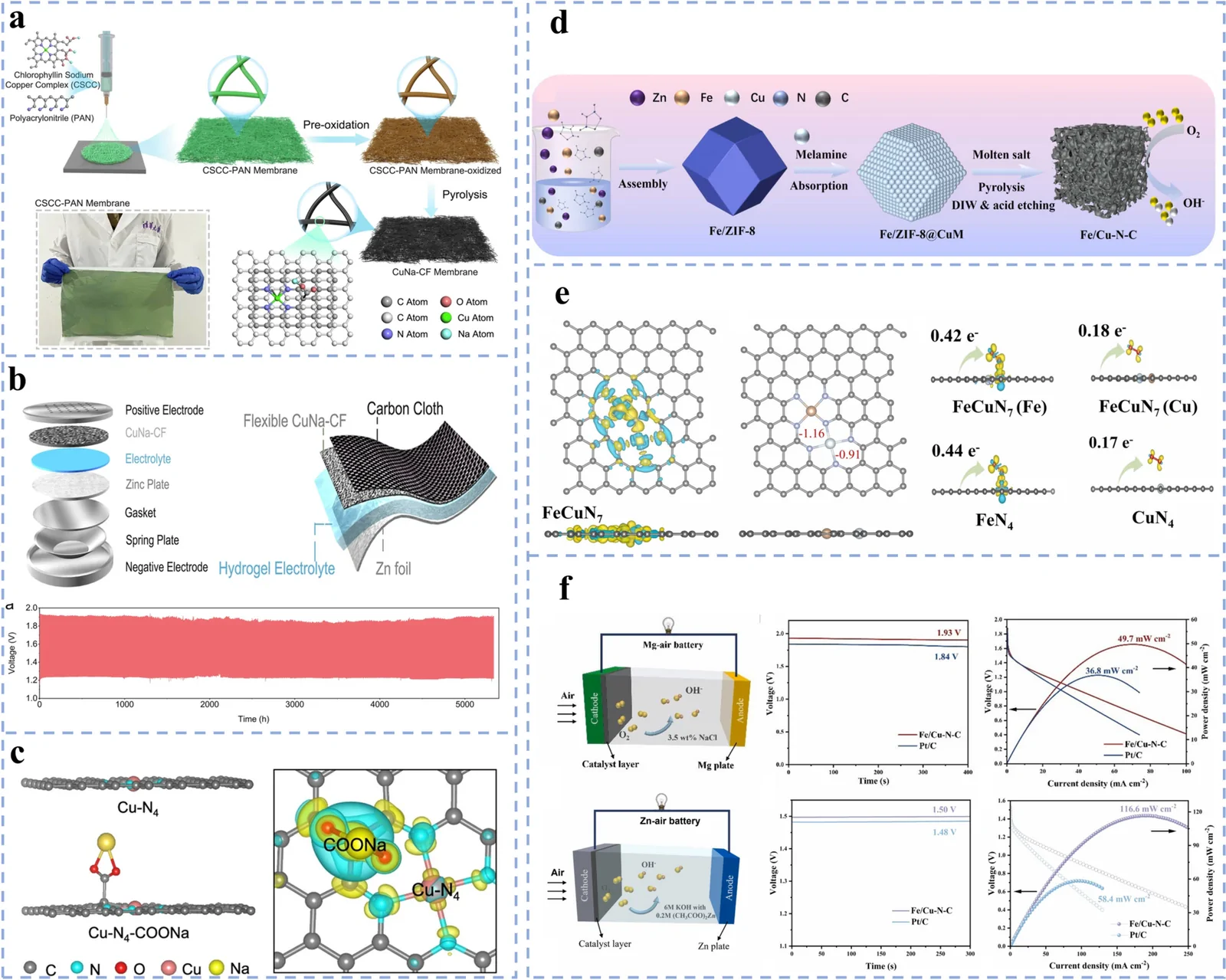
**a** Catalyst synthesis method. **b** Application of CuNa–CF in liquid and flexible all-solid-state zinc–air batteries. **c** Charge density difference analysis. **d** Schematic diagram of catalyst synthesis [173]. Copyright 2024, Springer Nature. **e** Charge density difference diagram and Bader charge analysis and state density of FeCuN_7_. **f** Application effect in Mg–air and Zn–air batteries [174]. Copyright 2024, Elsevier

The Li research team has developed an advanced catalytic system for metal–air batteries by strategically engineering a Cu–N–C catalyst through elemental regulation [173]. By incorporating sodium functional groups into the carbon framework to modify conventional Cu–N_4_ sites (Fig. 16a), they constructed a unique C_2_–Cu–N_4_–COONa coordination structure that synergistically enhances both catalytic activity and structural stability. This innovative configuration effectively modulates the electronic structure of Cu–N_4_ centers, optimizes the ORR pathway, and significantly mitigates carbon framework corrosion along with copper center migration and dissolution. When applied as an air cathode in zinc–air batteries (designated as CuNa–CF), the catalyst demonstrates exceptional overall performance (Fig. 16b), matching or even surpassing the ORR activity of commercial Pt/C catalysts while substantially improving battery longevity. The system maintains stable operation for over 5000 h at low current density and approximately 950 h at high current density. Atomic structure analysis and charge density difference mapping (Fig. 16c) further reveal that the Na–COO^−^ functional group induces electron redistribution around the Cu–N_4_ site, thereby optimizing O_2_ adsorption and intermediate conversion processes—a crucial factor in enhancing catalytic performance. Remarkably, the catalyst exhibits high power density and outstanding discharge characteristics in both flexible solid-state and button-type all-solid-state zinc–air batteries, indicating substantial potential for broad applications in wearable energy devices and micro-power sources.

In the design of TMCs, the modulation of active center electronic structures through main group elements has proved to be an effective strategy for enhancing catalytic performance and stability. Building on this approach, Zhang’s group developed a nitrogen-doped mesoporous carbon catalyst featuring well-defined Fe–N_4_ and Cu–N_4_ diatomic sites, synthesized via molten salt-assisted pyrolysis to achieve uniform metal distribution within the carbon matrix (Fig. 16d). In the resulting FeCu–N_7_ structure, Cu introduction induces asymmetric electron distribution around the Fe center, lowering O_2_ adsorption energy and preventing excessive OH* intermediate adsorption, thereby optimizing the rate-determining step of the ORR (Fig. 16e). Electronic structure analysis further reveals that Cu synergistically shifts the Fe d band center downward, weakening OH* binding strength and modulating the reaction pathway to enhance both activity and stability. Electrochemical tests (Fig. 16f) demonstrate that this diatomic catalyst significantly surpasses commercial Pt/C in magnesium–air and zinc–air batteries, robustly confirming the synergistic advantages of dual metal sites in improving electrocatalytic performance and durability for advanced energy storage systems.

The development of transition metal-based heterogeneous electrocatalysis systems for metal–air batteries has achieved remarkable progress, demonstrating significant enhancements in ORR activity, cycling stability, and energy conversion efficiency [175]. Through precise engineering of metal coordination environments, optimization of *d* orbital electronic configurations, and strategic implementation of defect engineering and interface coupling, substantial improvements have been realized in intermediate adsorption behavior and electron transfer kinetics. The synergistic integration of in situ characterization techniques with computational modeling has further illuminated the dynamic evolution of key intermediates and energy-determining steps, establishing a robust framework for understanding structure–activity relationships [176]. Despite these advances, fundamental challenges remain regarding the dynamic evolution of electrode–electrolyte interfaces, electrolyte-induced structural degradation, and the delicate balance between activity and durability. Future research directions should emphasize the regulation of interfacial charge distribution, investigation of active site dynamics under operational conditions, and development of well-defined catalytic architectures for stable integration into diverse device configurations. These developments represent crucial steps toward realizing high-energy–density, long-cycle-life metal–air battery systems for sustainable energy applications.

### Fuel Cells

**Fig. 17:**
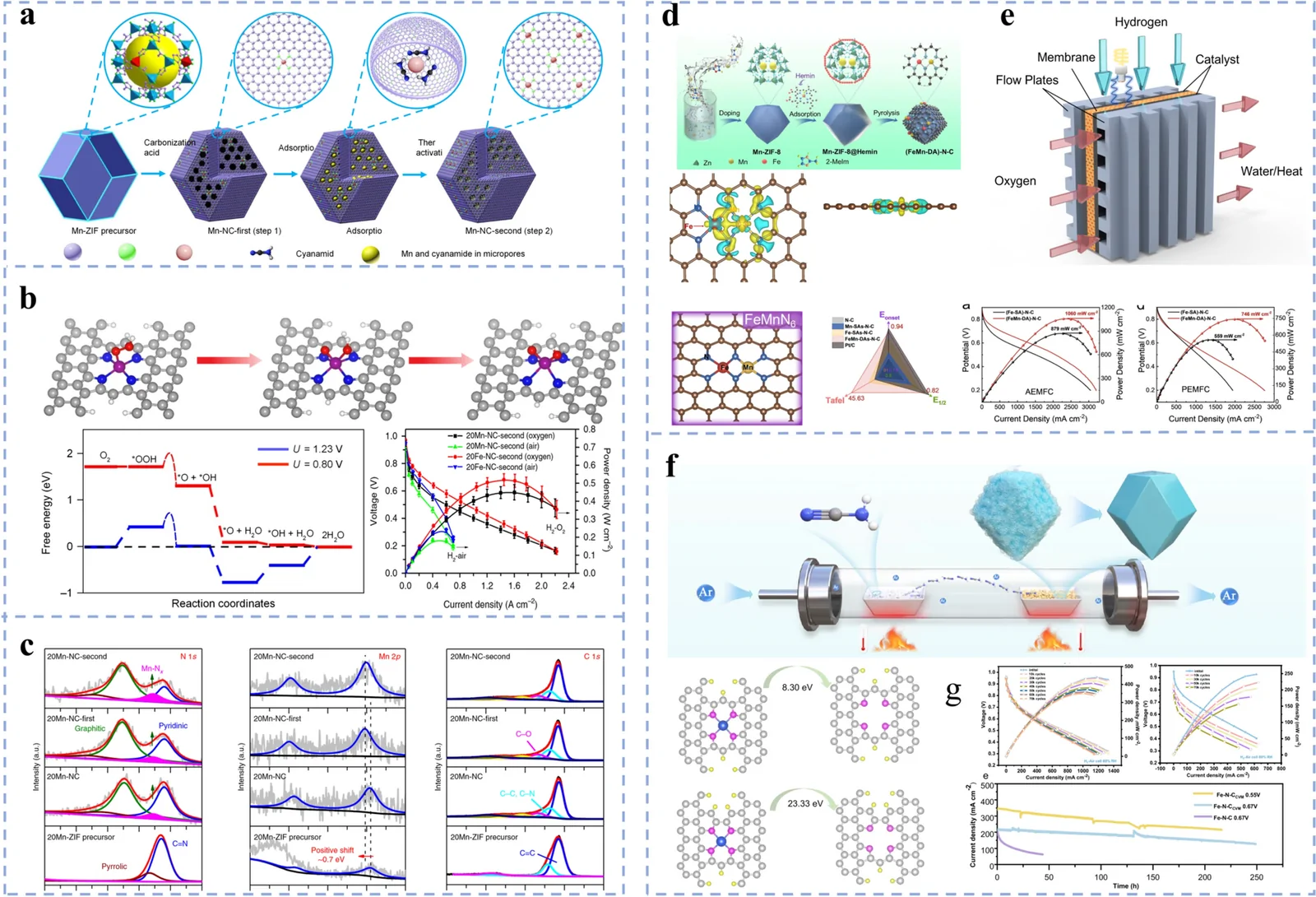
**a** Flowchart of the construction of the MnN_4_ active site catalyst with atomic-level distribution. **b** ORR free energy path of the MnN_4_–Cl_2_ site. **c** High-resolution XPS spectra of materials at different synthesis stages [177]. Copyright 2018, Springer Nature. **d** Synthesis path, electronic structure and three-dimensional radar map of FeMn co-doped N–C catalyst. **e** Schematic diagram of the application of the catalyst in proton exchange membrane fuel cells and anion exchange membrane fuel cells [178]. Copyright 2024, Springer Nature. **f** Schematic diagram of the synthesis of the Fe–N–CCVM catalyst and the S1-type FeN_4_ site can be converted into a N_4_C_12_ structure, while the FeN_2_ + N'_2_ site evolves into a N_4_C_11_ coordination environment. **g** Electrochemical test diagram of Fe–N–CCVM and the comparative material Fe–N–C [179]. Copyright 2024, Springer Nature

The high cost and scarcity of platinum-based catalysts have substantially hindered the large-scale commercialization of proton exchange membrane fuel cells, making the development of alternative TMCs a central focus in current fuel cell research. Addressing this challenge, Li et al. reported a proton exchange membrane fuel cell incorporating a highly dispersed manganese-based catalyst with exceptional stability (Fig. 17a) [177]. Through comprehensive characterization using in situ XPS, XANES, and EXAFS analyses, the researchers confirmed that manganese predominantly exists in the + 2 oxidation state with a well-defined Mn–N_4_ coordination structure, without detectable metal clusters (Fig. 17b, c). This Mn–N–C catalyst demonstrates superior performance in acidic electrolytes compared to conventional carbon-based nonmetallic catalysts, effectively facilitating the four-electron ORR pathway with a H_2_O_2_ yield below 2%, thereby minimizing the detrimental impact of by-products on fuel cell operation. Practical evaluation in membrane electrode assemblies reveals outstanding performance under reduced precious metal loading conditions and exceptional operational durability. The remarkable stability of the Mn–N–C catalyst in fuel cell applications is primarily attributed to the antioxidant properties of the MnN_4_ structure and its ability to suppress carbon support corrosion. Compared to iron and cobalt-based counterparts, manganese demonstrates superior resistance to Fenton reaction-induced catalyst degradation due to its lower reactivity with H_2_O_2_, thereby significantly enhancing fuel cell stability and operational lifetime.

Zhang’s research group successfully developed a bimetallic catalyst featuring adjacent Fe-based and Mn-based active sites for fuel cell applications (Fig. 17d) [178]. Electronic structure analysis revealed that Mn incorporation effectively modulates the electronic distribution of Fe centers, lowering the d band center position. This electronic optimization enhances OH* desorption capability and increases the reaction limiting potential, thereby significantly improving both the activity and stability of the ORR. When employed as a cathode material in fuel cells, the catalyst demonstrates exceptional performance—achieving high power density in both anion exchange membrane and proton exchange membrane fuel cells while surpassing commercial Pt/C catalysts in durability and methanol tolerance (Fig. 17e), highlighting its strong potential for practical energy conversion devices. In a complementary approach, Bai et al. achieved substantial improvements in Fe–N–C catalyst stability through chemical vapor modification (Fig. 17f) [179]. This process transformed conventional FeN_4_ sites into symmetric coordination structures containing both pyridinic and pyrrolic nitrogen atoms, while simultaneously enhancing carbon support graphitization, reducing structural defects, and establishing a more stable microenvironment that effectively suppresses Fe center displacement and structural collapse. Electrochemical evaluations (Fig. 17g) demonstrated that the modified catalyst exhibits accelerated reaction kinetics, reduced H_2_O_2_ yield (< 2%), and higher electron transfer number, along with remarkable durability—showing only 10% performance decay over 250 h at 0.67 V compared to > 70% decay within 43 h for unmodified counterparts. Furthermore, it achieved a peak power density of 450 mW cm^−2^ in proton exchange membrane fuel cells, representing a significant advancement toward practical implementation.

**Fig. 18:**
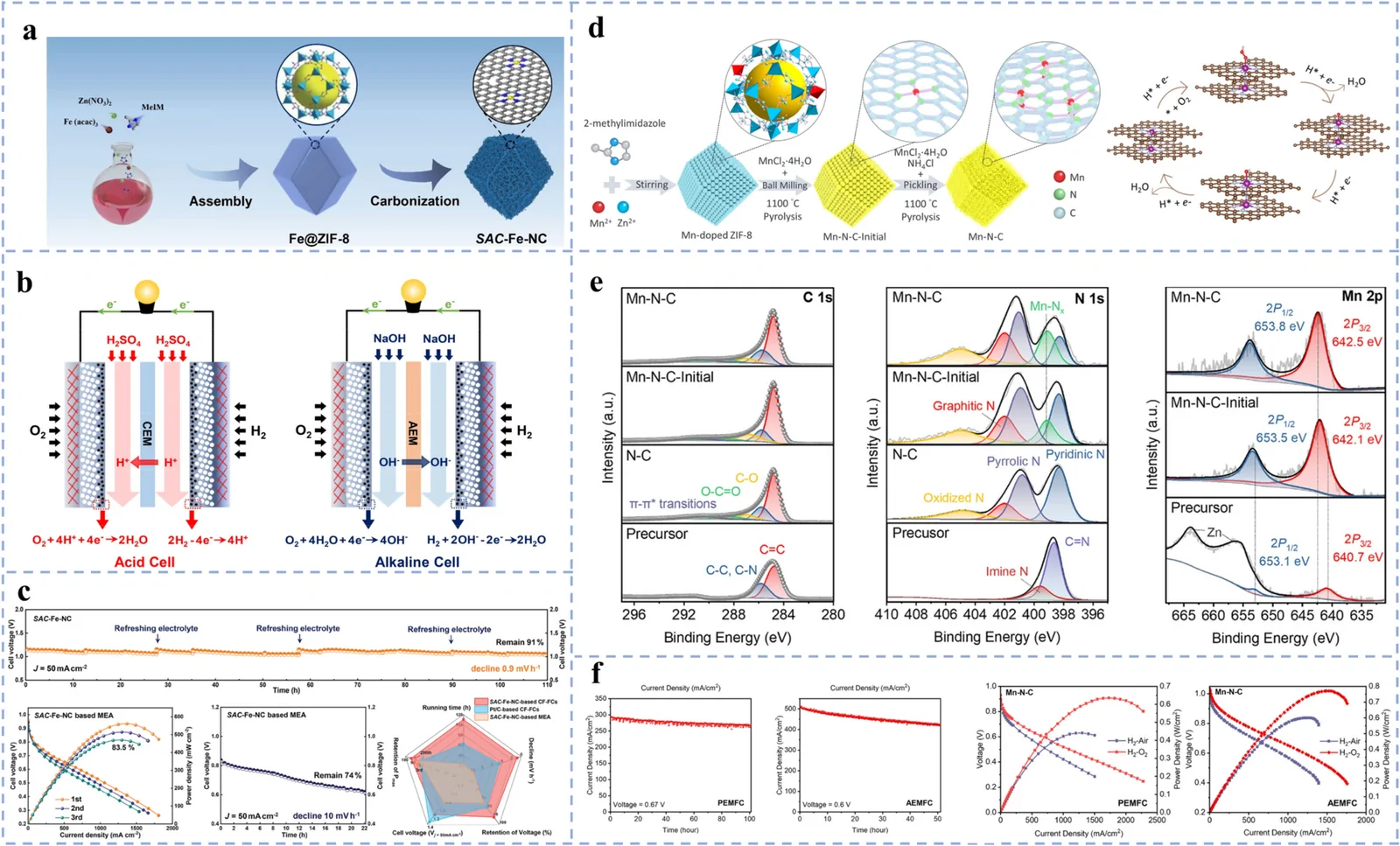
**a** Schematic diagram of the synthesis of SAC–Fe–NC from Fe@ZIF-8 after carbonization. **b** Acidic and alkaline CF–FC battery structures. **c** Battery performance and comparison of SAC–Fe–NC catalysts [180]. Copyright 2024, Wiley–VCH. **d** Schematic diagram of the synthesis of Mn–N–C catalysts and the adsorption configuration of intermediates during the ORR process. **e** C 1*s*, N 1*s* and Mn 2*p* XPS spectra of samples at different stages. **f** Electrocatalytic performance of Mn–N–C–4.5 in proton exchange membrane fuel cells and anion exchange membrane fuel cells [181]. Copyright 2025, Wiley–VCH

Shi’s research group developed an innovative acid–base coupled flow fuel cell (CF–FC) system that utilizes synergistic acidic ORR and alkaline hydrogen oxidation to harness additional electrical neutralization energy (Fig. 18a) [180]. Unlike conventional fuel cell configurations (Fig. 18b), this system employs acidic ORR at the cathode and alkaline hydrogen oxidation at the anode, separated by cation and anion exchange membranes to prevent direct neutralization while converting electrical neutralization energy into additional voltage output. This design increases the theoretical open-circuit voltage from 1.229 to 2.057 V. The team also designed a single-atom Fe–N–C catalyst with Fe–N_4_ sites as the cathode material, demonstrating exceptional ORR activity and four-electron pathway selectivity. In practical CF–FC operation (Fig. 18c), the catalyst achieved an output voltage of 1.16 V at 50 mA cm^−2^ with 91% voltage retention over 110 h and a minimal decay rate of 0.9 mV h^–1^, outperforming conventional membrane electrode assembly fuel cells and commercial Pt/C systems. The system maintained 83.5% of its initial performance through multiple charge–discharge cycles, with negligible Fe leaching, demonstrating excellent stability and practical potential. The battery module allows flexible power expansion through series, parallel, and hybrid configurations. In a separate development, Chen’s team created a Mn–N–C ORR catalyst with high-density atomically dispersed Mn active sites using a combined ball milling and dual high-temperature pyrolysis approach (Fig. 18d) [181]. This method promotes the formation of MnN_4_-rich sites and creates a distinctive axisymmetric dual MnN_4_ structure. The resulting material features well-defined mesoporosity, high graphitization, and uniform manganese–nitrogen distribution, exhibiting the lowest reaction energy barrier and enhanced dissolution resistance crucial for ORR activity and durability. XPS analysis revealed the evolution of elemental chemical states during synthesis (Fig. 18e), confirming that the three-step strategy effectively establishes Mn–N_x_ active sites while regulating carbon framework graphitization. The catalyst demonstrated exceptional ORR performance in both alkaline and acidic environments, achieving peak power densities of 649 mW cm^−2^ in proton exchange membrane fuel cells and 770 mW cm^−2^ in anion exchange membrane fuel cells. Accelerated aging tests showed only 18.4% performance degradation after 30,000 cycles, significantly outperforming Fe–N–C materials prepared by the same method and demonstrating superior oxidative corrosion resistance and long-term stability.

In fuel cell systems, performance is primarily governed by three critical factors: the coordination structure of metal active centers, the microstructural configuration of carbon supports, and the overall stability of catalytic materials [182]. At the material design level, enhancing electron transfer kinetics, improving reaction selectivity, and strengthening corrosion resistance can be achieved through precise regulation of metal–ligand bonding environments, optimization of carbon support graphitization and conductivity, and rational design of pore structure distribution. From the perspective of atomic-level regulation, key strategies for achieving high catalytic efficiency and durability include constructing highly dispersed single-atom active sites, suppressing metal agglomeration and undesirable side reactions, and steering the ORR toward the efficient 4e^−^ pathway. On the device integration level, developing novel electrode architectures compatible with diverse electrolyte environments presents promising avenues for enhancing both power output and operational stability. The advancement of fuel cell technology fundamentally relies on continuous innovation in high-stability, cost-effective catalytic materials, coupled with coordinated optimization across multiple scales—including material synthesis, interface engineering, and system integration—to ultimately realize efficient and sustainable energy conversion systems.

### ORR Pathway Selectivity for Electrochemical H_2_O_2_ Production

In both fuel cells and metal–air batteries, the ORR ideally proceeds through the four-electron pathway to achieve complete reduction of O_2_ to H_2_O (or OH^−^), ensuring high energy efficiency and device stability. However, the competing two-electron pathway that produces H_2_O_2_ is generally regarded as an undesired side reaction, as the generated peroxide species can lower energy output and corrode catalysts or carbon supports. Interestingly, this so-called undesired reaction in energy conversion devices can be advantageously harnessed in chemical synthesis [183]. The selective two-electron ORR pathway forms the electrochemical basis for sustainable H_2_O_2_ production, offering a green alternative to traditional anthraquinone processes. Therefore, transition metal-based heterogeneous catalysts that exhibit partial 2e^−^ selectivity in fuel cells or metal–air batteries can be repurposed as efficient catalysts for electrochemical H_2_O_2_ synthesis. This complementary relationship highlights how insights from energy conversion systems can guide the rational design of catalysts for selective oxygen reduction in chemical production.

**Fig. 19:**
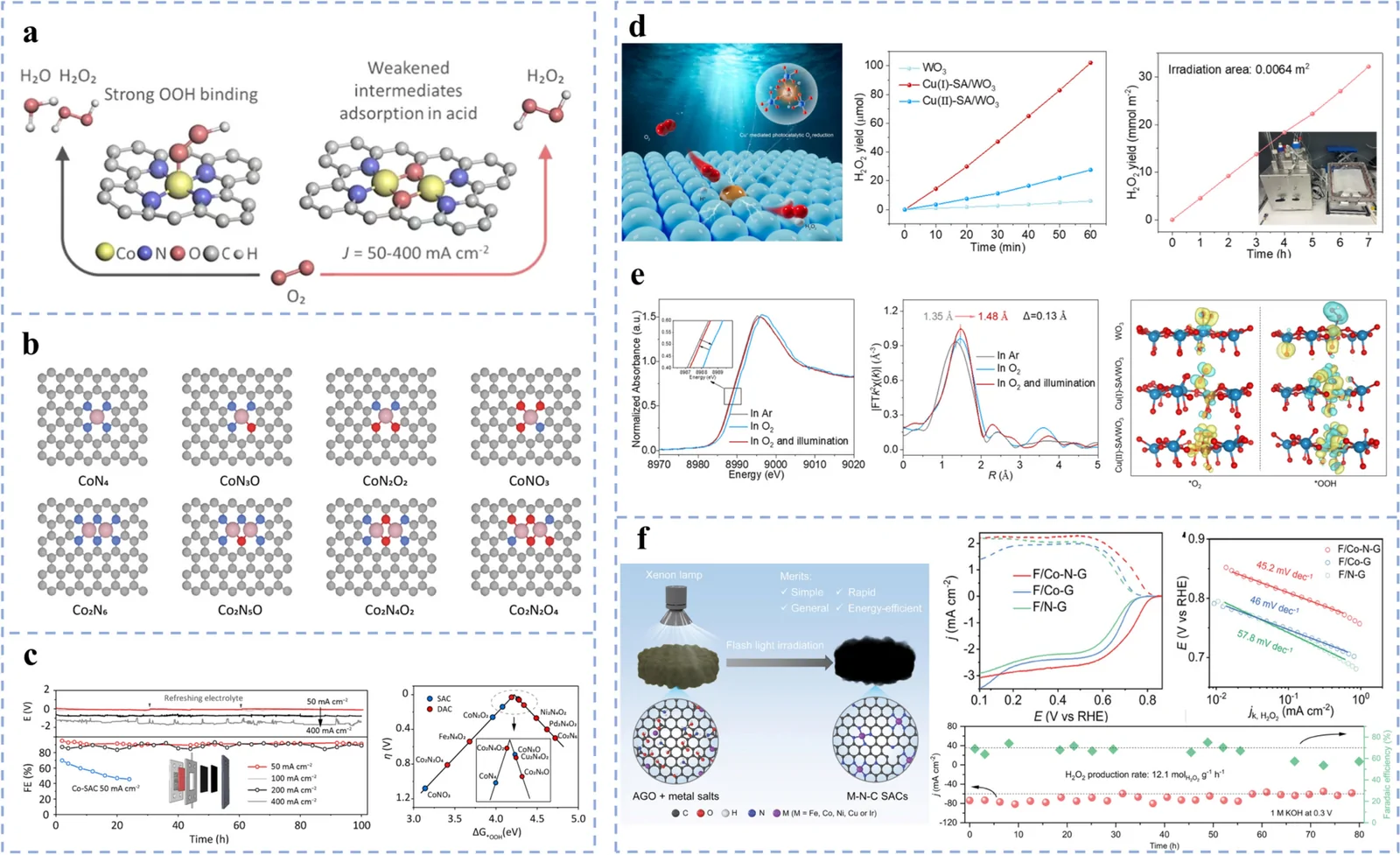
**a** Co–N_x_ site achieves highly selective H_2_O_2_ reaction mechanism by regulating intermediate adsorption under acidic conditions. **b** Stability of the catalyst at different current densities and volcano plot analysis. **c** Various Co–N/O coordination structure models used for calculation [184]. Copyright 2024, American Chemical Society. **d** Schematic diagram of photocatalytic O_2_ reduction promoted by Cu(I) single-atom and electrochemical test and actual reactor diagram. **e** In situ XANES and EXAFS spectra and the adsorption of intermediates on different catalyst surfaces and charge difference density maps during O_2_ photoreduction [185]. Copyright 2025, American Chemical Society. **f** Schematic diagram of flash irradiation-assisted synthesis of F/M–N–G SACs and electrochemical tests [186]. Copyright 2024, Wiley–VCH

Huang and colleagues successfully developed an efficient electrochemical system for synthesizing hydrogen peroxide in acidic media by constructing diatomic cobalt sites (Fig. 19a) [184]. The reaction mechanism was elucidated through schematic representation (Fig. 19b), demonstrating that the diatomic cobalt configuration significantly slows OOH* intermediate adsorption compared to conventional single-atom Co–N–C sites, thereby effectively inhibiting its further reduction to water. Electrochemical characterization (Fig. 19c) confirmed that the catalyst achieves over 95% H_2_O_2_ selectivity with a partial current density reaching 2.7 mA cm^−2^ in acidic electrolyte. When implemented in a flow electrolyzer, the system maintains high H_2_O_2_ production rate and stable operation over 100 h, substantially outperforming traditional Co–N–C catalysts. Subsequent investigations attribute this superior performance to the optimized electronic structure and reduced adsorption strength for oxygen intermediates enabled by the diatomic coordination environment.

Yang et al. [185] systematically investigated the photocatalytic synthesis of H_2_O_2_ using WO_3_^−^supported single-atom copper catalysts with controlled valence states. Their study compared the performance of WO_3_, Cu(II)–SA/WO_3_, and Cu(I)–SA/WO_3_ under visible light irradiation, providing the first detailed mechanism of O_2_ adsorption and activation at atomic Cu sites on WO_3_ surfaces. The results demonstrate that Cu(I) sites formed under low loading conditions significantly enhance photocatalytic activity, achieving an H_2_O_2_ production rate of 102 μmol h^−1^, 17.3 times higher than pristine WO_3_ (Fig. 19d). The catalyst maintained stable performance over 7 h of continuous operation in a panel reactor with linear H_2_O_2_ accumulation, indicating promising scalability for practical applications. Through a series of in situ XANES and EXAFS analyses (Fig. 19e), the research elucidated the electronic structure evolution and local coordination environment changes of Cu(I) sites during the reaction. Under O_2_ atmosphere with illumination, the average coordination bond length at Cu sites increased from 1.35 to 1.48 Å, confirming effective O_2_ activation following photogenerated electron capture. At Cu(I) active centers, the hydrogenation process of the key OOH* intermediate transitioned from endothermic to exothermic, substantially reducing the reaction energy barrier and enhancing selectivity toward the 2e^−^ ORR pathway. This electronic optimization enabled an exceptional photon quantum efficiency of up to 30%, representing a significant advancement in overcoming the efficiency limitations of conventional catalytic H_2_O_2_ synthesis methods.

For fundamental metal–nitrogen–carbon (M–N–C) catalysts, extensive research has confirmed their significant catalytic activity and remarkable stability in the 2e^−^ ORR. These materials are typically synthesized by incorporating transition metal centers and coordinating nitrogen atoms within a carbon-based framework. Gong’s team developed an efficient flash thermal-assisted strategy to rapidly achieve reduction, nitrogen doping, and metal atom anchoring at the atomic level, producing M–N–C single-atom catalysts such as Co–N_4_ [186]. This method is simple, rapid, energy-efficient, and applicable to various metal precursors, demonstrating the versatility of different central metal atoms in the electrochemical synthesis of H_2_O_2_. Building on this foundation, the study further compared the polarization curves and Tafel slopes of different catalysts in O_2_-saturated KOH electrolyte, all of which exhibited excellent ORR performance (Fig. 19f). This synthetic approach is equally applicable to the development of M–N–C single-atom catalysts based on other metals such as Fe, Ni, and Cu, demonstrating its universality and scalability while providing novel concepts and technical platforms for the green synthesis of H_2_O_2_ and the large-scale production of single-atom catalysts.

Transition metal-based heterogeneous electrocatalysis systems have demonstrated considerable potential for the electrocatalytic production of H_2_O_2_, though several challenges persist in achieving controlled selectivity, maintaining catalyst stability, and preventing compromised efficiency in industrial settings [187]. Recent research advances have addressed these limitations through innovative approaches including the construction of diatomic sites, introduction of π–d conjugated structures, and precise electronic structure optimization, collectively enhancing both catalytic activity and H_2_O_2_ selectivity. Concurrently, the adoption of rapid synthesis techniques such as photothermal methods has significantly improved preparation efficiency and material scalability. Future investigations should prioritize refined regulation of electronic structures and intermediate adsorption behaviors, enhancement of catalyst durability under acidic conditions and high current densities, and progression toward distributed, environmentally sustainable catalytic systems adaptable to diverse application scenarios (Table 1).

**Table 1:** Performance comparison of representative ORR electrocatalysts

Types	Electrocatalyst	Electrolyte	Durability	Half-wave potential (vs. RHE) (V)	Tafel slope(m V dec^−1^)	H_2_O_2_ yield	References
Fe	Fe SAs–HP	0.1 M KOH	> 30k cycles; 97.3% (50k s)	0.94	84.0	< 5%	[114]
	Fe_1_/DNC	0.1 M KOH	> 20 k cycles; 93% (50 h)	0.95	54.0	< 0.35%	[115]
	Fe_SA_–Fe_AC_/NC	0.1 M KOH	> 10 k cycles; 91% (10 h)	0.886	73	< 2%	[116]
	Fe–N–C_NH4I_	0.1 M KOH	> 20 k cycles; 96.7% (11 h)	0.924	52.1	< 1%	[117]
	N–FeN_4_	0.1 M KOH	100% (50 h)	0.91	64.9	< 1%	[118]
	FeNb/c-SNC	0.1 M KOH	> 5k cycles;	0.922	52	< 0.5%	[119]
	HEAC/Fe–NC	0.1 M KOH	> 20k cycles; 89% (40k s)	0.927	86	< 4%	[120]
	Fe–Mn DAC/N–C	0.1 M KOH	> 10k cycles; 93% (40 h)	0.91	58	< 3%	[178]
	Fe–N₄–CVM	0.1 M HClO_4_	> 30k cycles; 92% (100 h)	0.82	75	< 2%	[179]
	SAC–Fe–NC	0.5 M H_2_SO_4_	91% (110 h)	0.794	57.5	< 2%	[180]
Mn	Mn–pr–N–CG	0.5 M H_2_SO_4_	95% (20 h)	0.896	43.6	< 5%	[125]
	MnY–NC	0.1 M KOH	> 10k cycles; 100% (30 h)	0.93	58	< 3%	[126]
	MoP@Mn_SAC_–NC	0.1 M KOH	> 1000 cycles; 98% (10 h)	0.894	67.1	< 9%	[128]
	Mn_SA_/Mn_AC_–SSCNR	0.1 M KOH	> 5k cycles; 91% (42 h)	0.90	49.2	< 5%	[129]
	Mn, Fe–HCNS	0.1 M KOH	> 10k cycles; 91% (48k s)	0.87	65	< 6.6%	[171]
	Mn–γ-Fe_2_O_3_	0.1 M KOH	> 10k cycles; ~ 90% (40 h)	0.86	68	< 10%	[172]
	Mn–N–C	0.5 M H_2_SO_4_	> 30k cycles; 88% (100 h)	0.80	80	< 2%	[177]
Ni	FeCoNiMnCrO	1.0 M KOH	> 20k cycles;	0.87	62	< 5%	[138]
	Ni(OH)_2_/Pt	0.1 M HClO_4_	> 15k cycles;	0.912	65.8	< 4%	[140]
	Ni(OH)_2_–C_2_O_4_	0.1 M KOH	> 5k cycles; ~ 100% (20 h)	–	–	≈ 93%	[141]
	Ni–SAC/CNS	0.1 M KOH	100% (20 h)	–	79.5	≈ 89%	[137]
Co	Co(CN)_3_–Cub	0.1 M KOH	> 10k cycles	0.90	57	< 5%	[151]
	CoPc/CB–Mag	0.1 M HClO_4_	90% (100 h)	–	86	≈ 93%	[150]
	Co_2_–DAC	0.1 M HClO_4_	85%(100 h)	–	124	≈95%	[184]
	F/Co–N–G	0.1 M KOH	90% (20 h)	–	45.2	≈90%	[186]
Cu	Pt_1_–CuO_x_/Cu	0.1 M KOH	100% (100 h)	0.92	65	< 3%	[161]
	Cu–SA/SNC	0.1 M KOH	> 10k cycles	0.893	–	< 5%	[158]
	Cu–N–C	0.1 M KOH	> 10k cycles; 100% (50k s)	0.89	63	< 5%	[173]
	Cu/Fe–N–C	0.1 M KOH	> 5k cycles; 95% (50k s)	0.89	69.4	< 1.77%	[174]
Other	SmPO_4_	0.1 M KOH	> 5k cycles; 90% (12 h)	–	–	≈ 96%	[163]
	LaPO_4_	0.1 M KOH	> 5k cycles; 90% (12 h)	–	–	≈ 94%	[163]
	GdPO_4_	0.1 M KOH	> 5k cycles; 90% (12 h)	–	–	≈ 92%	[163]
	La–Cl SAs/NHPC	0.1 M KOH	> 6k cycles; 94.8% (30 h)	0.91	50.4·	< 4%	[164]
	FePc/Eu_2_O_3_	0.1 M KOH	> 20k cycles; 98.2% (100k s)	0.931	56.5·	< 3%	[165]
	Re–SAC	0.1 M KOH	> 10k cycles	0.89	72·	< 5%	[166]

## Summary and Outlook

### Summary

This review systematically summarizes the research progress of transition metal-based heterogeneous catalysts in the ORR, with emphasis on structure-dependent reaction mechanisms, electronic structure regulation, and catalytic design strategies. By systematically comparing Fe, Mn, Ni, Co, Cu, and rare earth-based systems, it elucidates the crucial roles of different metal centers in coordination environments, *d* orbital modulation, adsorption energy matching, and reaction pathway selection (2e^−^/4e^−^). Furthermore, the review highlights the synergistic effects of single-atom, dual-atom, and heterogeneous interface structures in modulating charge distribution, enhancing electron transport, and optimizing intermediate adsorption. Integrating in situ characterization with theoretical simulations, it also summarizes the mechanistic advantages of emerging strategies such as multimetal synergistic regulation, defect engineering, and high-entropy structures for improving catalytic activity and structural stability, and outlines their application prospects in fuel cells, metal–air batteries, and electrochemical H_2_O_2_ synthesis.

Despite significant breakthroughs achieved by transition metal-based catalytic systems in ORR research, several critical challenges remain, including structural reconstruction and dissolution under strong acidic or alkaline conditions, unclear adsorption–desorption kinetics of key intermediates, and the difficulty in balancing high activity with long-term stability. Future research should focus on atomic-level electronic structure design and multimetal synergistic regulation to construct highly stable heterogeneous structures with interfacial charge coupling and strain modulation capabilities. Moreover, the integration of in situ/operando spectroscopy with multiscale simulations is essential to uncover real reaction pathways and the dynamic evolution of active sites. The synergistic advancement of material design, mechanistic understanding, and system optimization will provide theoretical foundations and technical guidance for developing the next generation of efficient, durable, and low-cost ORR electrocatalysts.

### Outlook

Future research on the oxygen reduction reaction is expected to progress from atomic-level regulation toward multidimensional, system-level engineering, in which activity, selectivity, and durability must be optimized simultaneously. Beyond static electronic structure descriptors, it is essential to elucidate how coordination environments, charge distribution, and intermediate adsorption evolve dynamically under operating conditions, particularly in acidic media and at high current densities. Under such extreme conditions, structural reconstruction, metal dissolution, and site aggregation remain critical challenges for transition metal heterostructures, especially those based on single-atom and dual-atom motifs. Addressing these issues requires mechanistic understanding of active site stability and adaptability under strong interfacial electric fields, continuous redox cycling, and intense mass transport, rather than treating stability as an intrinsic materials property.

**Fig. 20:**
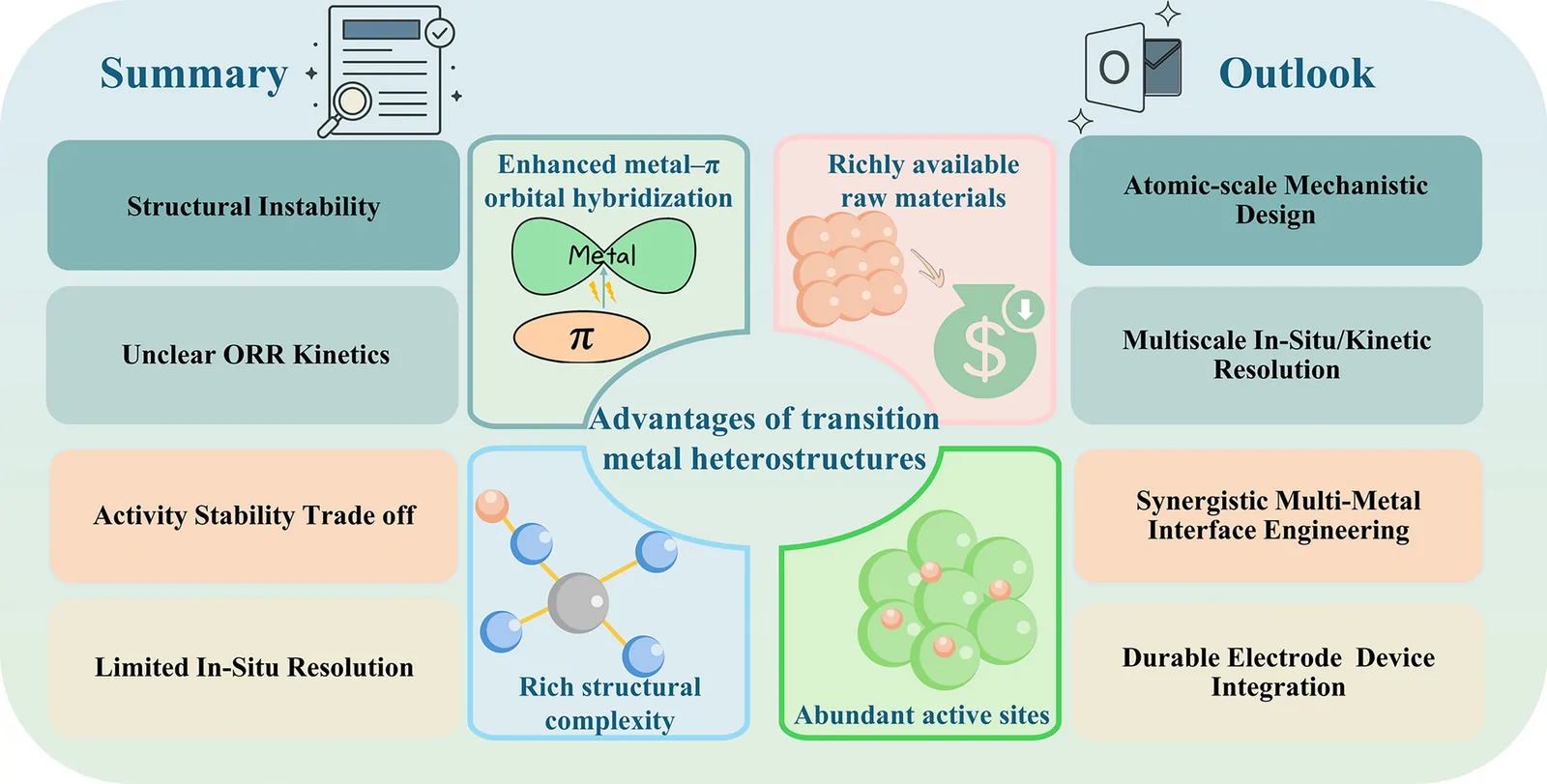
Challenges and prospects of ORR in heterogeneous transition metal materials

Selective regulation of two-electron versus four-electron ORR pathways represent another key frontier, especially for hydrogen peroxide electrosynthesis. Future advances will likely rely on multifield coupled control strategies, in which coordination asymmetry, interfacial electric fields, solvation structures, and spin-related effects collectively reshape reaction pathway branching. In this context, the increasing complexity of heterogeneous design highlights the need for data-driven and machine learning-assisted approaches to explore high-dimensional structure–property–kinetics relationships. From a translational perspective, bridging the gap between atomic-level precision and industrial implementation will further require synthesis strategies compatible with high-throughput processing, structural reproducibility, and cost constraints, together with electrode architectures that integrate catalyst stability with efficient mass and charge transport. Overall, future progress in ORR catalysis will depend on the co-development of mechanistic insight, scalable materials design, and device-level integration, enabling the transition from laboratory demonstrations to practical clean energy technologies (Fig. 20).
